# OmpW-mediated sensing of MUC2 GalNAc contributes to the ileal tropism of *Salmonella* Typhi

**DOI:** 10.1016/j.celrep.2026.117801

**Published:** 2026-08-17

**Authors:** Chang-Kyu Yoon, Gi Young Lee, Chanmi Kim, Katharina Ribbeck, Jeongmin Song

**Affiliations:** 1 Department of Microbiology and Immunology, Cornell University, Ithaca, NY, USA; 2 Department of Biological Engineering, Massachusetts Institute of Technology, Cambridge, MA, USA; 3 Present address: Department of Microbiology, Gachon University College of Medicine, Incheon, South Korea; 4 Present address: Department of Molecular Biology, Jeonbuk National University, Jeonbuk, South Korea; 5 Lead contact

## Abstract

*Salmonella* Typhi (STy), the causative agent of typhoid fever, preferentially infects the ileum, but the basis for this preference has remained unknown. We show that STy exploits MUC2-derived monosaccharides, such as *N*-acetylgalactosamine (GalNAc), to upregulate invasion and target the ileum—opposite to the response of closely related *S.* Typhimurium (STm), whose invasion is inhibited by MUC2. Three STy-specific extracellular residues of the outer-membrane protein OmpW (K141/T142/Q193) engage GalNAc, and a periplasmic relay through the short FepE_STy, RS12510, and TonB transmits this signal to activate the master invasion regulator HilD. Mice lacking MUC2 or gut microbiota lose STy ileal tropism, which is restored by oral GalNAc-containing monosaccharides. An OmpW triple-mutant STy strain establishes a murine oral STy infection model, identifying STy-MUC2 glycan interactions as an early step of typhoid pathogenesis and a potential target for prevention.

## In brief

*Salmonella* Typhi (STy) causes typhoid fever by preferentially invading the ileum, yet the molecular basis of this tropism has remained unknown. Yoon et al. show STy senses microbiota-released mucin sugars via the surface protein OmpW to trigger invasion, and leverage this mechanism to establish an oral mouse model of typhoid.

## Graphical Abstract

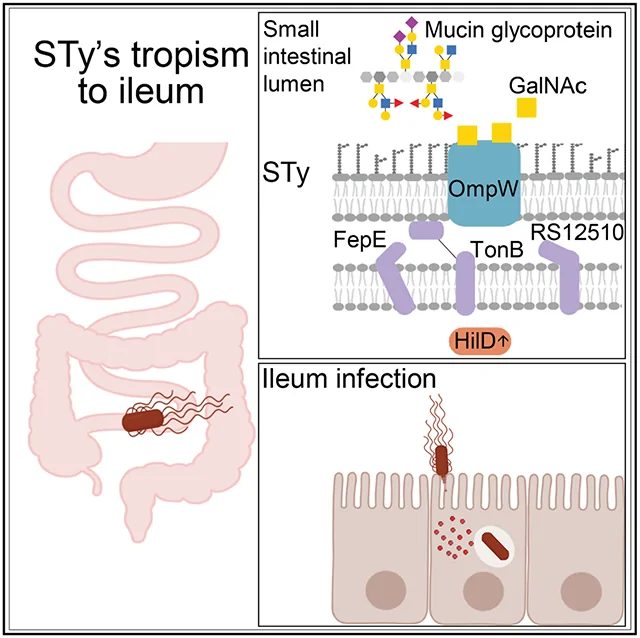

## INTRODUCTION

*Salmonella enterica* serovar Typhi (*S.* Typhi, STy) causes typhoid fever, which is estimated to have infected 9.3 million people and claimed more than 100,000 deaths globally in 2021.^1^ Typhoid fever vaccines are available and provide partial protection.^2,3^ However, the emergence and spread of multidrug-resistant (MDR) and extensively drug-resistant (XDR) strains of STy pose a significant threat to public health.^4-6^ Advancing our understanding of STy pathogenesis could give valuable insights into developing new treatments against antibiotic-resistant cases of this life-threatening systemic disease. Both STy infection and reinfection are predicted to begin in the small intestine, predominantly in the ileum, based on colonoscopic findings in typhoid patients with intestinal lesions.^3,7-9^ In humans, ileal preference not only links to effective STy systemic infection but is also associated with ileal perforation, a deadly complication of STy infection.^3,7-9^ However, the underlying mechanism behind STy ileum preference remains poorly understood.

Although STy and *S. enterica* serovar Typhimurium (STm) share approximately 89% of their genomes, they cause strikingly distinct diseases in humans.^10,11^ STm has a broad host range and typically remains localized to the intestinal mucosa, producing a self-limiting, neutrophil-rich gastroenteritis.^12,13^ STy, in contrast, is a human-specific pathogen that breaches the intestinal epithelium with limited overt inflammation, traffics through mononuclear phagocytes, and establishes a systemic, often persistent infection of the reticuloendothelial system, including the liver, spleen, and gallbladder.^3,13^ This dichotomy reflects extensive adaptive evolution of STy, including acquisition of the Vi capsule, the typhoid toxin, modulation of TLR-flagellin recognition by TviA, and serovar-specific reading-frame variations in surface and virulence factors.^14-18^ Productive systemic infection requires efficient initial invasion across the follicle-associated epithelium of the distal small intestine, where Peyer’s patches are concentrated and where M cells provide a preferential portal of entry for *Salmonella*.^19^ The ileum-biased entry of STy is predicted to be, therefore, mechanistically linked to its broader virulence program of intracellular dissemination, whereas STm, which causes gastroenteritis, distributes more uniformly along the small intestine. However, little is known about how STy reads ileal cues to selectively launch this program.

A defining feature of the small-intestinal environment is the mucus layer, which is produced by goblet cells and dominated by the gel-forming mucin MUC2.^20,21^ MUC2 has a heavily *O*-glycosylated protein backbone consisting of proline/threonine/serine-rich tandem repeats decorated with branched *O*-glycans that can account for up to ~80% of its mass and that protect the polypeptide from proteolysis while serving as a hydrated barrier against luminal contents.^20-23^ These *O*-glycans are built on a core *N*-acetylgalactosamine (GalNAc) attached to serine or threonine and elaborated with galactose (Gal), *N*-acetylglucosamine (GlcNAc), fucose, and sialic acids; GalNAc, Gal, and GlcNAc collectively constitute the dominant glycan mass of intestinal MUC2.^22,23^ Although the small intestine has a thinner and more discontinuous mucus layer than the colon, MUC2-derived glycans are abundant along its length and are continuously released through host turnover and microbial glycohydrolase (GH) activity, generating a steady pool of free monosaccharides at the epithelial interface.^20,24^ Beyond serving as a physical barrier, mucin glycans are increasingly recognized as bioactive signals that dampen virulence and biofilm programs in *Pseudomonas aeruginosa*, *Streptococcus mutans*, *Candida albicans*, *Vibrio cholerae, and Citrobacter rodentium*,^22,25-28^ and were shown to suppress *Salmonella* pathogenicity island 1 (SPI-1)-mediated invasion in STm.^23,29^ Yet *Salmonella*-mucin interactions are not uniformly inhibitory: mid-twentieth-century experiments and subsequent reports observed that mucin pre-exposure paradoxically enhanced STy virulence in mice, although the molecular basis remained unresolved.^30,31^ STy and STm also encode chitinase and related glycoside hydrolases that remodel the host glycome at the epithelial surface,^32,33^ indicating that mucin glycans are an active, two-way interface between host and pathogen. Together, these observations frame an unresolved question: whether and how STy uses MUC2-derived carbohydrates to direct its preference for the ileum.

Here, using epithelial cell cultures and murine infection models, we demonstrated that STy exploits the primary glycan components of MUC2, a major cue in the small-intestinal environment, to enhance its infectivity and its preference for the ileum.

## RESULTS

### MUC2 is a cue promoting *S.* Typhi infectivity

MUC2-derived glycans are major intestinal and biliary tract glycans,^20^ suggesting a potential role of MUC2 glycans in STy infection biology in the small intestine. MUC2 has been identified as a factor that reduces the virulence of STm.^23^ Despite the high genomic similarity of 89% between the two strains, STm typically causes self-limiting gastroenteritis in humans.^10,11^ Previous studies have shown that STy, when pre-exposed to mucin, enhanced its infectivity in mice, although the underlying mechanism remained unclear.^30,31^ We infected human epithelial Henle-407 cells with STy or STm pre-cultured in high-salt LB (LB.3) with or without 0.2% porcine MUC2 (used here as a substitute for human MUC2),^23^ and quantified invasion by colony-forming unit (CFU) assays. We observed a marked increase in STy invasion following pre-exposure to MUC2, compared to STy cultured in LB.3 without MUC2 (Figure 1A). Consistently, immunofluorescence microscopy using an anti-STy antibody also indicates that MUC2 serves as a cue promoting STy invasion (Figures 1B and 1C). In contrast, we observed that MUC2 inhibited STm invasion (Figures 1D-1F), consistent with existing literature that highlights MUC2 as an inhibitory factor for STm and other bacterial strains^22,23,25-27^ and thereby validating the distinct response of STy to MUC2. Since motility is crucial for *Salmonella* infectivity in the gut,^34^ we also investigated whether MUC2 alters *Salmonella* motility by conducting soft agar motility assays. We found that MUC2 increased STy motility while decreasing STm motility (Figures 1G and 1H). Notably, the growth of *Salmonella* in LB.3, with or without 0.2% MUC2, remained comparable (Figure S1), indicating that MUC2 acts as both an activator of STy and an inhibitor of STm, thereby regulating their infectivity.

To understand how STy responds to MUC2, we conducted comparative RNA sequencing. We found that MUC2 enhances STy infectivity at the transcriptional level (see Figures 1I and 1J). Specifically, we observed increased expression of 82 genes in STy (Figures 1I and 1J; Table S1). The majority of these genes were associated with the Type 3 secretion system (TTSS) SPI-1 (24 genes) and flagella assembly, motility, and chemotaxis (24 genes) (Figures 1K-1N). SPI-1 is a well-known virulence factor that contributes to *Salmonella* infectivity, while flagella are crucial for efficient infection.^35,36^ The upregulation of SPI-1 and motility genes in response to MUC2 suggests the involvement of key transcription factors, such as HilD, HilC, and RtsA for SPI-1 genes, as well as the FlhDC complex for motility and chemotaxis genes (Figure S2). In contrast, consistent with the inhibitory effect of MUC2 on STm infectivity (Figures 1D-1H), we found decreased mRNA expression of TTSS and motility genes in STm in response to MUC2 (see Figures 1M and 1N). The RNA sequencing results were validated using RT-qPCR (see Figures 1O and 1P). Collectively, these results indicate that the regulation of *Salmonella* infectivity by MUC2 occurs at the transcriptional level.

### The main MUC2 glycan components are essential in enhancing *S.* Typhi infectivity

GalNAc, Gal, and GlcNAc are the predominant glycan components of MUC2, which are present in an approximate ratio of 3:3:1^22,23^ (Figure 2A; Figure S3). To investigate whether these glycan components enhance STy infectivity, we conducted invasion assays. STy and STm were cultured in LB.3 containing one of the following monosaccharide mixtures (MMs): MM1 (GalNAc:Gal:GlcNAc at 3:3:1), MM2 (2:2:1), MM3 (1:1:1), and MM4 (1:1:2). We found that MM1 and MM2 significantly enhanced STy infectivity, while MM3 and MM4 resulted in decreased infectivity. This suggests that GalNAc and Gal are essential for the increased infectivity mediated by MUC2 glycan components, whereas GlcNAc does not play a crucial role (Figure 2B). In contrast, the infectivity of STm decreased in response to the MUC2 glycan component mixtures (Figure 2B), which further emphasizes the distinct responses of STy and STm to MUC2 and its glycan components. Importantly, these results demonstrate STy’s remarkable ability to differentiate among GalNAc, Gal (the 2-deacetylated counterpart of GalNAc), and GlcNAc (the C4 epimer of GalNAc), which are otherwise structurally near-identical (Figure 2C). Specifically, when cultured in LB.3 containing GalNAc or Gal, STy showed increased invasion efficiency, whereas GlcNAc reduced its infectivity (Figure 2D). This behavior was not observed with STm (Figure 2D).

### STy cannot import GalNAc

*Salmonellae* are unable to transport GalNAc,^37^ whereas they can transport and utilize Gal and GlcNAc as sole carbon sources. This suggests that the different reactions to GalNAc likely originate on the bacterial surface. Consistent with the literature, we confirmed that, unlike *E. coli* BL21, which can transport GalNAc,^37^ STy ISP2825 and STm SL1344 cannot transport or utilize GalNAc as a sole carbon source (Figure 2E). Through mutagenesis studies, we further demonstrated that the genes STy *galP* and *mglB* are essential for the transport of Gal, while STy *nagE* and *manX* are crucial for the transport of GlcNAc (Figure 2F; Figure S4). Although these mutants still showed enhanced STy infectivity mediated by GalNAc, the effects mediated by Gal and GlcNAc were reduced in the corresponding mutants. This supports the prediction that GalNAc-mediated effects initiate at the bacterial surface (Figure 2G).

### The surface protein OmpW is crucial for the GalNAc-mediated enhanced infectivity

We reasoned that an outer-membrane protein with an extracellular (EC) domain likely discriminates among GalNAc, Gal, and GlcNAc at the cell surface to drive the STy-specific response. To identify candidates, we examined the surface proteins of STm.^38^ Using this STm dataset and the STy genome, we conducted a comparative bioinformatics analysis that identified 72 surface proteins in STy (see Figure 3A; Table S2). Among these, 17 surface proteins in STy differ in their amino acid sequences within the EC domains compared to their counterparts in STm (Figure 3A). This suggests that at least one of these 17 surface proteins is responsible for the differing responses of STy and STm to GalNAc.

To prioritize candidates, we filtered our RNA-seq dataset for proteins with RPKM values of 100 or higher, narrowing the list to four STy surface proteins: OmpW, OmpF, LamB, and FljB (Figures 3A and S5; Table S2). To test whether these proteins underlie the MUC2- and GalNAc-mediated upregulation of STy virulence, we generated four STy chimera strains in which the open reading frame (ORF) of each candidate was replaced with its STm counterpart, leaving the STy ISP2825 regulatory background otherwise intact. In this design, any GalNAc-dependent phenotype that correlates with the chimera assignment can be attributed to differences in EC-domain residues.

All four chimeras attenuated the glycan-driven upregulation of STy virulence, with OmpW and FljB contributing most strongly (Figure 3B). RT-qPCR analysis of *hilD* expression in these chimeras is in line with STy infectivity results (Figure S6). The quadruple chimera was no more impaired than the OmpW single chimera, indicating that the four proteins act non-synergistically and that OmpW is the dominant determinant (Figure 3B). To gain insights, we further generated and examined each STy gene-deletion mutant and found that deleting *ompW* decreased STy infectivity in response to GalNAc, whereas deleting any of the remaining three genes increased STy infectivity relative to the *ΔompW*-GalNAc response (Figure 3C). Unlike the well-characterized FljB (the sole STy flagellin, homologous to both STm FljB and FliC), LamB (a maltose/maltodextrin transporter), and OmpF (a porin), OmpW is a poorly understood outer-membrane protein containing both EC and periplasmic domains (PDs) (Figure 3D; Figure S5). STy and STm OmpW differ at three EC-domain residues: K141/N141, T142/A142, and Q193/H193 (Figure 3D). To assess how these residues affect *Salmonella* virulence, we also created STm strains expressing OmpW from STy. We found that STm OmpW from STy behaves like STy, as it increased infectivity in response to MUC2 and GalNAc (Figure 3E). Additionally, we evaluated the relative importance of these three residue differences in STy infectivity by generating mutants, STy OmpW^K141N/T142A^ and STy OmpW^Q193H^. Both mutants failed to enhance STy infectivity in response to MUC2 and GalNAc (Figure 3F), indicating that the three EC-domain residues of OmpW in STy are crucial for this response.

### The short FepE, RS12510, and TonB are involved in OmpW-mediated enhanced infectivity of *S.* Typhi

We generated a reporter strain, *S.* TyphiP*_hilD_*’*lacZ*, to identify factors that link OmpW-GalNAc recognition to HilD activation, and used it for a genome-wide transposon-insertion sequencing (TNseq) screen of an ~50,000-mutant library (averaging ~10 insertions per gene). TNseq recovered 28 candidate genes, and subcellular localization analysis narrowed these to three membrane proteins with PDs: FepE_STy, RS12510, and RS17905 (Table S3). Subsequent mutagenesis confirmed roles for the short FepE (FepE_STy) and RS12510 (Figures 3G-3J; Figure S7). Notably, STy expresses a short FepE of 78 amino acids rather than the full-length 339-amino-acid form found in *S.* Paratyphi. As a result, *fepE*_STy is currently annotated as a pseudogene^10^ (see Figure 3H; Figure S8).

In *S.* Paratyphi, the full-length FepE is responsible for controlling the length of the *O* antigen.^39^ However, the short FepE in STy lacks the long PD that is essential for this function (Figure S8), indicating that FepE_STy has a role that is specific to STy. STy*ΔfepE* failed to induce *hilD* promoter activity (Figure S7) and to enhance STy infectivity in response to GalNAc (Figure 3I). Interestingly, this phenotype could be rescued by introducing the short FepE, but not by the full-length FepE (Figure 3I). This suggests that short *fepE*_STy is specifically involved in linking OmpW-GalNAc to HilD activation, thereby increasing STy infectivity.

RS12510 is an uncharacterized periplasmic protein consisting of 237 amino acids and includes an N-terminal transmembrane domain (Figure S9). STy strains lacking RS12510 (STy*ΔRS12510*) failed to induce *hilD* promoter activity and did not enhance STy infectivity in response to GalNAc (Figure 3J; Figure S7). Complementation with RS12510 successfully rescued this phenotype (Figure 3J). In contrast, STy*ΔRS17905* remained responsive to GalNAc, suggesting that RS17905 is likely a false positive result from the TNseq screen (Figure 3I; Figure S7).

TonB, an inner-membrane protein with an extended PD, has not been characterized in STy. In other bacteria, TonB’s primary and best-characterized function is to couple the proton-motive force at the inner membrane to TonB-dependent receptors at the outer membrane, thereby energizing the active transport of iron-siderophores, vitamins, and other substrates.^40^ TonB also interacts with outer-membrane proteins to transmit signals into the cytoplasm.^41^ We investigated whether STy *tonB* (comprising 242 amino acids) is involved in enhancing STy infectivity in response to GalNAc. Our findings revealed that STy*ΔtonB* failed to enhance STy infectivity in this context, and this phenotype could be rescued by *tonB* complementation (Figure 3K).

We further examined whether their PDs are necessary for the increased STy pathogenesis mediated by OmpW by creating mutants lacking the C-terminal PD, specifically STy*fepE^ΔC^*, STy*RS12510^ΔC^*, and STy*tonB^ΔC^*. We observed that these mutants lacking the PD did not show increased infectivity in response to GalNAc (Figure S10). These results indicate that although the detailed pathway remains to be explored, including whether they interact physically or only functionally, FepE_STy, RS12510, and TonB are essential for the increase in STy pathogenesis mediated by the OmpW K141/T142/Q193-GalNAc interaction (Figure 3L).

### OmpW-mediated responses to MUC2-related monosaccharides are linked to STy ileum preference

Both MUC2, which enhances STy infectivity (this study), and bile, which reduces STy infectivity,^16^ are present in both the biliary and intestinal tracts. To distinguish where the MUC2-mediated mechanism is most relevant, we tested STy responses to MUC2 glycan components across a range of bile concentrations (1–4%). Bile is far more concentrated in the gallbladder than in the small intestine, whereas MUC2 glycans are comparable at both sites.^42^ We found that MUC2 glycan components, specifically GalNAc and Gal, still increased STy infectivity in the presence of 1–2% bile (Figure 4A). However, this effect was less pronounced at 4% bile (Figure 4A), suggesting that STy’s response to MUC2 glycan components likely plays a crucial role in the small intestine but a minor role in the gallbladder.

To explore the role of STy responses to MUC2 glycan components in its infection biology within the small intestine, we orally gavaged sex- and age-matched littermates of Cmah null mice with either STy or STm (Figure 4B). Cmah null mice express “human-type” glycan receptors and are an established model for STy that resolves virulence contributions from typhoid toxin and the Vi capsule.^15,16,43^ One day after infection, we performed CFU assays to assess the *Salmonella* burden in three regions of the small intestine: the duodenum, jejunum, and ileum.^44^ We observed that STy shows a preference for the ileum in mice (Figure 4C), consistent with reports of a similar preference in humans.^3^ In comparison, STm colonized the ileum as previously demonstrated,^19^ but unlike STy, STm had a greater presence in the proximal segment of the small intestine, specifically the duodenum and jejunum (Figure 4C).

Next, we investigated whether the interaction between GalNAc and OmpW is critical to STy’s preference for the ileum using *Salmonella* OmpW chimera strains. We found that STy expressing OmpW from STm behaves like wild-type STm. In contrast, STm expressing OmpW from STy behaves like wild-type STy (Figure 4D), indicating that the OmpW-GalNAc interactions are significant for STy’s ileum preference. Consistently, the ileum preference was not observed in Muc2 null mice^45,46^ (Figure 4E). The absence of this preference in Muc2 null mice was reversed by oral gavage with MM1, a mixture resembling MUC2 glycan components, but not with the control mixture MM4 (Figure 4F). Intestinal microbiota produce GHs and carbohydrate-activated enzymes that degrade mucin and host-derived glycans as nutrient sources.^24^ Since STy lacks mucin-specific GHs and has limited mucin-degrading enzymes^32,33^ (Figure S11), we next examined whether STy can exploit microbiota-degraded MUC2 glycan components.

We pre-treated mice with streptomycin to eliminate major murine gut microbiota.^47^ Forty-eight hours after treatment, we infected the mice with either STy or STm and conducted CFU assays one day post-infection (Figure 4G). Control mice were pretreated with PBS. Notably, the invasion preference of STy in the distal small intestine was no longer observed in streptomycin-pretreated mice, as STy colonized all parts of the small intestine with a higher bacterial burden (Figure 4H). Similarly, STm did not exhibit a preference for the proximal small intestine; rather, it showed a modest preference for the distal part in streptomycin-treated mice (Figure S12). We then orally gavaged the antibiotic-pretreated mice with either MM1 (resembling MUC2 glycan components) or MM4 (control). Much like the results from Muc2 null mice, the supplementation of MM1 to streptomycin-pretreated mice partially restored the ileum preference of STy, while MM4 did not (Figure 4I). These findings indicate that STy’s responses mediated by OmpW to MUC2 glycan components—mainly accessible through the action of intestinal microbiota—significantly contribute to its preference for the ileum.

### *S.* Typhi expressing OmpW_STm_ serves as a promising model for studying oral STy infection

Several murine models have been developed for STy research,^16,48-51^ but none have successfully demonstrated productive oral STy infection. Consequently, the STm orogastric model is commonly used as a surrogate for studying STy infection.^52,53^ The STy OmpW_STm_ strain, which carries the mutations K141N, T142A, and Q193H, mimics STm’s infection in the small intestine (Figure 4D). This prompted us to investigate whether STy OmpW_STm_ causes increased systemic infection in mice. If confirmed, this could establish a murine oral infection model for studying specific aspects of systemic STy infection.

We orally infected Cmah null mice with STy, STy expressing OmpW_STm_, and STm. Three days post-infection, we measured bacterial burden in the liver and spleen using CFU assays (Figure 5A). Our results showed that STy-expressing OmpW_STm_ had significantly higher CFUs in both the liver and spleen compared to those infected with STy (Figure 5B). Additionally, we observed that splenic melanosis occurred more frequently in mice infected with STy-expressing OmpW_STm_ than in those infected with STy (Figure S13). In contrast to the oral infection studies, we did not observe OmpW-mediated effects on bacterial burden in the liver and spleen when the bacteria were administered intraperitoneally (IP) (Figure 5C). For IP infection experiments, we used a hypervirulent capsule STy strain as previously established.^15,16,43^ These findings highlight the role of OmpW in the small intestine and suggest its potential utility in developing a murine oral infection model.

This work, along with previous studies, indicates that *Salmonella*-host interactions in the small intestine differ between STy and STm. To determine whether the STy OmpW_STm_ animal model can reveal differences in these interactions, we examined STy, STy expressing OmpW_STm_, and STm in littermates of Nlrc4 null mice.^54-58^ The Nlrc4 protein is involved in regulating STm infection by sensing components of TTSS and flagella components.^54-58^ We orally infected Nlrc4 null mice with STy, STy expressing OmpW_STm_, and STm, and then performed organ CFU assays 3 days post-infection (Figure 5A). At this early stage of systemic infection, when macrophage Nlrc4-mediated effects on bacterial burden were not detectable,^59^ we did not observe increased systemic infection of STy OmpW_STm_ in the livers and spleens of Nlrc4 null mice, unlike with STm (Figure 5D). To determine whether Nlrc4 in intestinal epithelial cells plays a significant role in this phenotype, we performed *ex vivo* CFU assays using primary epithelial cells isolated from Cmah null and Nlrc4 null/Cmah-null mice. These intestinal epithelial cells were infected with STy, STy expressing OmpW_STm_, and STm. Our results showed that Nlrc4 expressed in intestinal epithelial cells plays an essential role in OmpW-mediated regulation of STy virulence (Figure S14). This finding highlights the distinct roles of Nlrc4 during the early stages of STy and STm infections, underscoring the advantage of using STy OmpW_STm_ over STm in murine oral infection studies.

## DISCUSSION

This work demonstrates that STy uses predominant MUC2-derived monosaccharides to activate virulence and target the ileum, a poorly understood aspect of STy pathogenesis. The determinants we identified span multiple compartments: OmpW in the outer membrane; the short FepE_STy, RS12510, and TonB in the periplasm/inner membrane; and HilD in the cytoplasm. Together, these define a multi-compartment signal-relay model rather than a single lock-and-key event (Figure 3L). Notably, the closely related STm responds to the same monosaccharides by downregulating infectivity, highlighting adaptive divergence in STy.

Among four surface/outer-membrane proteins linked to STy/STm response differences (OmpW, OmpF, FljB, LamB), only *ompW* deletion decreased STy infectivity in response to GalNAc; deleting any of the remaining three genes increased infectivity relative to the *ΔompW*-GalNAc response (Figure 3C). This suggests that most bacteria, including STy, dampen infectivity in response to GalNAc via a yet-to-be-defined mechanism, and that STy has acquired three OmpW EC substitutions that instead enhance MUC2/GalNAc-driven infectivity; consistently, mutating these residues abolished the response. We therefore propose that the three STy-specific residues (K141, T142, Q193) constitute the initial site of GalNAc recognition, and that engagement alters OmpW’s interactions with downstream periplasmic partners.

We identify three periplasmic partners for OmpW-mediated STy infectivity in response to MUC2 and GalNAc: FepE_STy, RS12510, and TonB. The strict requirement of their PDs for the GalNAc response (Figure S10) is explained by a signal-relay role, in which a conformational or energetic state change at the OmpW outer surface is propagated through periplasmic contacts to converge on the enhanced STy infectivity. However, whether they interact—physically or functionally via other molecules—warrants further investigation.

Little is known about each periplasmic partner in this role. FepE_STy is thought to be nonfunctional due to a stop codon at amino acid position 79. However, this study reveals that FepE_STy is the result of adaptive evolution and contributes to a newly identified mechanism underlying STy’s infectivity and ileal preference. We demonstrated that the full-length FepE encoded by other bacteria, which regulates LPS *O*-antigen length, cannot substitute for the specific role of FepE_STy, indicating the unique adaptation of STy virulence factors for controlling virulence in the small intestine. TonB’s canonical role is energizing TonB-dependent outer-membrane receptors for iron-siderophore, vitamin, and solute transport,^40^ but earlier *Salmonella* studies also cast it as a signal-transducing element linking outer-membrane occupancy to the cytoplasm.^41^ Our data do not distinguish an energizer role from a pure signaling role, but place TonB at the relay step between OmpW and HilD; further investigations are required to distinguish these possibilities. RS12510, a functional periplasmic partner in the signal relay, is an uncharacterized protein with a short N-terminal α-helical domain predicted to be in the inner membrane and a long periplasmic C-terminus region. HHpred analysis predicted that the C terminus region is homologous to the enzymatic domain of L-alanyl-D-glutamate peptidase (Figure S9). Given this predicted peptidase function, RS12510’s mode of action in the signal relay is expected to differ quite significantly from that of the two other periplasmic partners.

MUC2 also coordinately induces flagellar/chemotaxis and SPI-1 invasion genes (Figures 1I-1N), consistent with the extensive cross-regulation of the flagellar (FlhDC) and SPI-1 (HilD/HilC/RtsA) regulons in *Salmonella* that deploys motility and invasion together at the epithelial surface.^34-36^ Our TNseq screen for *hilD*-activating loci recovered both periplasmic relay components (FepE_STy, RS12510) and flagellar/motor genes (Table S3), and our gene-swap screen recovered the flagellin FljB (Figures 3A and 3B). Two observations place the major flow of information as OmpW → periplasmic relay → HilD: the OmpW K141N/T142A and Q193H mutants collapse the MUC2/GalNAc transcriptional response (Figure 3F), and *ΔompW* abolishes the relay whereas *ΔfljB* still shows GalNAc-enhanced infectivity (Figure 3C). We therefore propose that GalNAc engagement at OmpW is relayed through FepE_STy/RS12510/TonB to HilD, whose induction of FlhDC and SPI-1 deploys motility and invasion together; whether FljB is a downstream output or an alternative input remains an open question.

Because MUC2 is the predominant glycoprotein in both the biliary and intestinal tracts, we asked where the GalNAc signal operates. Bile dose-dependent assays showed that GalNAc-mediated induction diminishes at higher bile concentrations, positioning MUC2 glycans as signals in the gut rather than in the gallbladder. Consistently, mice lacking MUC2 or gut microbiota lost infectivity induction and ileal preference, and oral MUC2-derived monosaccharides restored ileal tropism (Figures 4E-4I), underscoring GalNAc’s role in STy small-intestinal biology. In MUC2 *O*-glycans, GalNAc occupies the innermost, peptide-proximal position (α-linked to Ser/Thr as the Tn epitope). GalNAc sensing in the small intestine could therefore reflect two non-exclusive sources: free monosaccharides liberated from MUC2 by mucin-degrading microbiota, as our microbiota-depletion and MM1 rescue experiments support (Figures 4H and 4I), and inner glycan layers exposed by partial degradation or remodeling of the mucus, yielding truncated *O*-glycans with terminal GalNAc.

A related mechanistic question raised by our *in vivo* findings is why a MUC2-driven enhancement of invasion would manifest as a preference for the ileum rather than as uniformly elevated colonization throughout the small intestine. Oral supplementation of MM1 partially restored ileal tropism in both Muc2-null and microbiota-depleted mice (Figures 4F and 4I), even though the administered monosaccharide mixture would be expected to be available throughout the small intestine. This argues against a simple spatial-gradient model in which more GalNAc is delivered to the ileum than to the duodenum or jejunum and instead suggests that the ileum is the location at which an increase in invasion capacity is most consequential. Several features of the ileum are likely to contribute: the ileum harbors the highest density of follicle-associated epithelium and M cells of the small intestine, which serve as principal portals for *Salmonella* translocation^19^; the distal small-intestinal microbiota is more abundant and more actively mucin-degrading than the proximal microbiota, providing the proximate pool of accessible MUC2-derived monosaccharides^20,24^; and bile acid concentrations fall along the small intestine, which—given our finding that high bile attenuates the GalNAc response (Figure 4A)—creates a permissive window for the OmpW-mediated program in the distal segments. We therefore favor a model in which invasion is rate-limiting for STy expansion, specifically in the ileum, so that any cue that boosts invasion (such as MUC2/GalNAc sensing) is readout preferentially as enhanced ileal colonization. Experimental dissection of this missing link—for example, by selectively enabling or disabling invasion only at defined intestinal positions, or by mapping the spatial distribution of STy invasion events in real time—is a possible direction for future work.

Notably, we found that an OmpW triple mutant of STy, similar to that of STm, induces systemic infection in mice, particularly via the natural oral route. Our findings indicate that NLRC4 in intestinal epithelial cells is crucial for OmpW-mediated regulation of STy virulence, highlighting the significant role of STy-MUC2-related monosaccharide interactions in the small intestine during the early stages of infection. This suggests that STy expressing an OmpW mutant could serve as a promising model for studying the early (first few days) stages of oral STy infection, thereby enabling further investigation of host-pathogen interactions and how *Salmonella* breaches the gut barrier to cause systemic infection.

Together, our findings resolve the long-standing mystery of STy ileal tropism and reveal mechanisms of small-intestinal infection that offer potential targets for preventing and treating antibiotic-resistant typhoid fever.

### Limitations of the study

This study has several limitations. First, to find out the signal at the monosaccharide level, we used GalNAc, Gal, and GlcNAc. We note that these monosaccharides are not unique to MUC2. Second, although microbiota depletion abolished STy’s ileal preference and oral MM1 partially restored it (Figures 4H and 4I), we did not identify which specific members of the small-intestinal microbiota release MUC2-derived monosaccharides at the relevant time and place. Defining this minimal mucin-degrading community—and whether *O*-glycan release and monosaccharide distribution are gradient-distributed along the duodenum-jejunum-ileum axis—will require complementary gnotobiotic colonization and targeted metagenomic/glycomic approaches. Third, our experimental systems rely on surrogates for the human small intestine: Henle-407 cells and Cmah-, Muc2-, and Nlrc4-null murine models. Neither fully recapitulates human intestinal cellular composition, mucus architecture, nor microbial ecology. Primary human ileal enteroids with a defined glycan-degrading consortium would be a natural next step. Porcine MUC2, used here as a surrogate, differs from human MUC2 in fucosylation, sialylation, sulfation, tandem-repeat number, and core glycan structures.^23,60,61^ Because our mechanism is driven primarily by monosaccharides (GalNAc/Gal/GlcNAc) rather than by full-length MUC2, we expect the core finding to be generalizable, but testing in a fully human MUC2 context warrants further investigation. Fourth, at the biochemical level, we have not yet demonstrated direct binding of GalNAc to OmpW, nor have we mapped the periplasmic physical or functional interactions among OmpW, FepE_STy, RS12510, and TonB. The signal-relay model that best fits our genetic data will require further investigation. Finally, the STy *ompW*_STm_ oral-infection model captures the early intestinal stage of STy systemic disease (the first day or first few days), but, like all current murine STy models, does not reproduce long-term systemic infection; conclusions drawn from this model should therefore be interpreted as informing early invasion biology rather than the full arc of STy pathogenesis.

## STAR★METHODS

### EXPERIMENTAL MODEL DETAILS

#### Mammalian cell culture conditions

Human intestinal epithelial Henle-407 cells were cultured in DMEM high glucose (Corning, cat# 10-017-CV) supplemented with 10% FBS (Phoenix Scientific, cat# PS-100). Cells were kept at 37°C in a cell culture incubator with 5% CO_2_. Cells were maintained below 80% confluence and routinely tested for mycoplasma.

#### Mouse lines and husbandry

All animal experiments in this study were approved by the Cornell IACUC (protocol number 2014-0084). C57BL/6J, human glycan receptor-expressing Cmah null mice (Jackson Laboratory, RRID: IMSR_JAX:017588), Muc2 null mice, and Nlrc4 null mice were used for oral gavage or intraperitoneal infections of *S.* Typhi and *S.* Typhimurium. Mice were bred in-house and maintained in specific pathogen-free (SPF) ECRF animal facilities; infection experiments were conducted in an ABSL-2 environment in the TMCF facility. Both facilities follow a 10–14-h light–dark cycle (lights on at 5 a.m., off at 7 p.m.), 65–75°F (18°C–24°C), and 30–70% humidity. Food and water (neutral pH) were provided *ad libitum*. Genotypes were routinely confirmed by PCR with specific primers (Table S4), with Sanger sequencing also performed for Nlrc4-null mice.

#### Bacterial strains and culture conditions

*S*. Typhi ISP2825 and *S*. Typhimurium SL1344 were used as parental strains and were cultured in LB (Miller) or LB containing 0.3 M NaCl (LB.3) unless otherwise indicated. Note that when MUC2 or its related monosaccharides were added, LB.3 was buffered with 10 mM potassium phosphate (pH 6.7). Where indicated, 50 mg/mL kanamycin, 20 mg/mL tetracycline, or 80 mg/mL 5-bromo-4-chloro-3-indolyl-β-D-galactopyranoside (X-gal, Goldbio, cat# 7240-90-6) was added to the media. For glycan uptake tests (Figures 2E and 2F), we used minimal media [5 mM KCl, 7.5 mM (NH_4_)_2_SO_4_, 0.5 mM K_2_SO_4_, 1 mM KH_2_PO_4_, 10 μM MgCl_2_, 0.005% tryptophan, and 50 mM Tris/MES (2-(N-morpholino)ethanesulfonic acid), pH 7.4]^62^ supplemented with indicated carbon sources: galactose (Sigma, cat# 59-23-4), *N*-acetylgalactosamine (GalNAc) (Cayman, cat# 1811-31-0), and *N*-acetylglucosamine (GlcNAc) (Sigma, cat# 7512-17-6).

## METHOD DETAILS

### Bacterial mutant generation

Details of primers, strains, and plasmids used in this study are listed in Tables S4 and S5. Plasmids were constructed by Gibson assembly using in-house enzyme mix (T5 exonuclease, NEB cat# M0363S; Phusion polymerase, NEB cat# M0530S; Taq DNA ligase, NEB cat# M0208L).^15,63^ The open reading frame (ORF) deletion mutants of *S*. Typhi and *S*. Typhimurium were generated as previously described.^15^ Briefly, approximately 600-bp DNA fragments upstream and downstream of each ORF were cloned into NotI/BamHI-double-digested pSB890, a suicide vector carrying *sacB*. The resulting plasmids were transformed into *E. coli* β2163Δ*nic*35 and used for conjugation to *Salmonella* strains. Conjugants were induced to have two single-crossover events by placing them sequentially under positive selection pressure (resistance to tetracycline) and then negative selection pressure (sensitivity to sucrose). *S*. Typhi chimera and point mutant strains were generated by using the same strategy. Each ORF carrying point mutations was inserted into the corresponding sites of each pSB890-derived plasmid. The resulting pSB890 plasmids were transformed into *E. coli* β2163Δ*nic*35 strains and conjugated them into each ORF deletion mutant of *S*. Typhi or *S.* Typhimurium, as indicated in the figures. Mutations on the chromosomes of STy and STm mutants were confirmed via PCR and/or Sanger sequencing.

To construct the *fepE*, RS12510, and *tonB* complementation strains, each gene was PCR-amplified and linked to the *rpsM* promoter by cloning into EcoRI/KpnI-double-digested pWSK-P_*rpsM*_-mCherry plasmid. *S*. Typhi *fepE* carries a stop codon at amino acid position 79. The short allele 1–79 (*fepE*^short^) was amplified from *S*. Typhi, and full-length *fepE* (378 aa) was amplified from the *S*. Typhi *fepE*^+^ strain, in which the stop codon at position 79 (TAG) has been changed to TGG. The pWSK-derived expression plasmids were transformed into *S.* Typhi via electroporation (Bio-Rad, Gene Pulser Cuvette, cat# 165–2088 and Gene Pulser Xcell Electroporation Systems cat# 1652660). For promoter activity assays, *S*. Typhi reporter strain was constructed by replacing K3U68_01740 (GenBank: CP080960.1, nucleotide positions at 407,015 to 412,401) with *E. coli* LacZ transcriptionally fused with the *S*. Typhi *hilD* promoter via pSB890-based homologous recombination, resulting in *S*. Typhi P_*hilD*_-LacZ. A *lacZ* gene was amplified from pXD70LacZ1-promoterless-LacZ (Addgene plasmid # 191617; RRID: Addgene_191617).^64^ No growth defect was observed in these strains when cultured in LB. Strains were verified via Sanger sequencing available through the Cornell Institute Biotechnology Resource Center (BRC) Genomics Facility (RRID: SCR_021727).

### Porcine MUC2 preparation

Two batches of purified MUC2 were used, resulting in comparable results in our study. The first batch of purified MUC2 was obtained from the Ribbeck Lab (MIT). The second batch was prepared in the Song Lab (Cornell). Briefly, MUC2 was purified from porcine intestine as previously described.^26,27,65,66^ Six porcine intestine samples from 2-week-old male pigs were dissected. Small intestines (total length 3.2–3.8 meters) were cut into pieces of ~15 cm and squeezed to remove food waste, then gently washed with 70% EtOH and PBS. Approximately 26.6 g of scrapings per sample was obtained from the inner mucus layer using a tongue scraper. The samples were solubilized (1 g of scrapings per 5 mL) in 10 mM phosphate buffer, pH 7.0, 0.2 M sodium chloride, 5 mM EDTA, protease inhibitors (Thermo Scientific, cat# A32965), and 0.04% sodium azide (Capitol Scientific, cat# 1953-57). Cellular debris and remaining food waste were removed by centrifugation at 8,000 × g for 30 min at 4°C. The samples were centrifuged at 50,000 × *g* for 1 h at 4°C (Beckman Coulter Avanti JXN-26), and the supernatant was filtered through an Amicon Ultra-50 100K MWCO filter (Merck, cat. # UFC50100). This was followed by gel filtration on a Sepharose CL-2B (Sigma, cat. #65099-79-8) column packed into an XK 16/20 (Cytiva, 28-9889-37). The glycoproteins were prepared from the fractions within the bed volume, then desalted and freeze-dried. MUC2 was reconstituted in LB.3 medium at a concentration of 0.2% by incubating at 4°C for 24 h before experiments.

### Bacterial growth assay

*S*. Typhi ISP2825, *S.* Typhimurium SL1344, and *E. coli* BL21(DE3) strains were cultured in LB medium overnight. After inoculating from an overnight culture at a 1:40 dilution, *Salmonella* strains were grown in LB.3 medium buffered with 10 mM potassium phosphate buffer (pH 6.7) until OD_600_ reached approximately 1.0. For the growth test in minimal medium, bacterial cells cultured in LB.3 were washed twice and then incubated in minimal medium without any carbon sources for 30 min. The strains were cultured in a minimal medium containing 0.2% of each glycan as the sole carbon source in a 96-well microplate (Falcon, cat# 353072). OD_600_ was measured every 30 min using a Tecan Infinite M Plex (Tecan, RRID: SCR_026522). For bacterial growth assays in LB.3 containing MUC2, the culture was serially diluted 10-fold at each incubation time, spread onto LB.3 agar plates, and the colony-forming units (CFUs) were counted. The CFU counts were used to calculate the total number of bacterial cells in the culture.

### Bacterial invasion assay

Henle-407 cells were seeded at 10^5^ cells per well in 24-well plates and incubated overnight. In some cases, a coverslip was placed on the cell culture plates before the cells were seeded for imaging intracellular bacteria. *S.* Typhi and *S.* Typhimurium strains carrying the pWSK-mCherry plasmid were grown in a buffered LB.3 medium containing glycans or MUC2 for ~3 h until their OD_600_ reached approximately 1.0. The cells were infected with *S.* Typhi or *S.* Typhimurium in Hanks’ Balanced Salt Solution (Corning, cat# 21-020-CV) at a multiplicity of infection (MOI) of 30 and 15, respectively, for 1 h. The cells were washed with PBS and incubated in DMEM containing 100 μg/mL gentamicin (Lonza, cat. #7-518Z) for 30 min to eliminate extracellular bacteria. For CFU determination at 2 h post-infection, the infected cells were incubated for 30 min in DMEM media containing 10 μg/mL gentamicin, washed with PBS, lysed in 1 mL of PBS with 0.1% sodium deoxycholate for 15 min at room temperature, and 100 μL of a 10-fold serially diluted lysate was spread on LB agar plates. For immunofluorescence assays, the coverslips were fixed in PBS containing 4% paraformaldehyde (PFA) for 10 min at room temperature, washed with PBS to quench the PFA, and nuclei were counterstained with 4′,6-diamidino-2-phenylindole (DAPI) (Invitrogen, cat# D3571) for 15 min at room temperature. Rabbit anti-O9 antibody (SSIDiagnostica, cat# REF40276, 1:200) or rabbit anti-O4 antibody (Hardy Diagnostics, cat# M10311, 1:100) was used for detecting *S.* Typhi and *S.* Typhimurium, respectively. Intracellular *Salmonellae* were imaged using a BZ-X810 microscope (Keyence), with the Plan Fluorite 20× LDPH (BZ-PF20LP) objective used to acquire 100 images covering an area of 5.290 mm × 3.968 mm of each coverslip. For Figure 3C, Henle-407 cells were seeded at 1 × 10^5^ cells per well in 24-well plates and incubated overnight. *S.* Typhi and mutant strains were cultured overnight in Miller LB medium. The seed culture (40 μL) was inoculated into 2 mL of LB containing 0.3 M NaCl and 10 mM potassium phosphate buffer (pH 6.7), with and without 0.2% GalNAc. Bacteria grew to an OD600nm of 1 (~3 h). Cells were infected with *S.* Typhi or mutant strains in HBSS (Thermo Fisher cat# 14025092) at an MOI of 30 for 1 h. After infection, cells were washed with PBS and incubated with DMEM (Corning cat# 45000-312) containing 100 μg/mL gentamicin for 30 min to kill extracellular bacteria. Cells were then washed with PBS and incubated in DMEM containing 10 μg/mL gentamicin for 30 min. Infected cells were lysed in 1 mL of PBS containing 0.1% sodium deoxycholate for 15 min at room temperature, and 100 μL of a 10-fold serially diluted lysate was spread on LB agar plates.

### Image quantification of intracellular bacteria

Intracellular *S.* Typhi and *S.* Typhimurium in phalloidin-stained Henle-407 cells were quantified using the BZ-X810 image analyzer (Keyence) as previously described.^15^ For each image, the bacterial count was normalized to the number of DAPI-stained nuclei to control for cell density, and each data point in Figures 1C and 1F represents the normalized value from a single image. *n* = 120 images per condition were collected from three independent biological experiments.

### Swarming motility test

*Salmonella enterica* serovar strains were cultured with or without MUC2 as described above. Cells were diluted 1/100 (final OD_600_~0.01) and spotted on LB.3 plates containing 0.25% agar. The diameters of *S.* Typhi and *S.* Typhimurium colonies were measured after incubation at 37°C for 24 and 16 h, respectively, because typhoidal serovars are less motile than *S.* Typhimurium. The diameter was calculated assuming that the halo’s curvature approximates a circle,^14^ using the iBright CL1500 imager (Invitrogen, RRID: SCR_026565).

### Bacterial RNA sequencing

*S.* Typhi or *S.* Typhimurium cells were cultured in LB.3 supplemented with or without 0.2% purified porcine MUC2 for 3 h at 37°C. After fixing the cells in 100% cold methanol for 1 h at −20°C, the pellet was washed with RNAprotect Bacteria Reagent (Qiagen, cat. #76506), followed by lysis in TE buffer (10 mM Tris-HCl, 1 mM EDTA, pH 8.0) containing lysozyme (1 mg/mL). RNA extraction was performed using the Qiagen RNeasy Mini Kit (Qiagen, cat. #74104) with in-column DNase digestion (Qiagen, cat. #79254). The RNA concentration was measured using the Qubit RNA HS kit (Invitrogen, cat. #Q32852), and RNA quality was assessed by agarose gel electrophoresis.

Bacterial RNA sequencing was performed as previously described with minor modifications.^43^ Briefly, 4 μg of total RNA was hybridized with in-house oligomer mixes specific to *S*. Typhi rRNA (71 oligomers, 7 μM each, Table S4) and digested with thermostable RNase H (NEB, cat #M0523S) for 30 min at 60°C, followed by DNase I digestion (DNase I-XT, NEB, cat #M0570S) for 30 min at 37°C. Note that in-house oligomers were designed by using the Oligo-ASST program.^67^ cDNA libraries were prepared using the Maxima H Minus Double-Stranded cDNA Synthesis Kit (ThermoFisher, cat #K2561) with random hexamers. The purified libraries were end-prepped with the Ultra II End-prep mix (NEB, cat #E7546L), barcoded (ONT, cat # EXP-NBD104687), and pooled for adaptor ligation (ONT, cat # EXP-NBD104). RNAclean XP beads (Beckman Coulter, cat # A63987) and AMPure XP beads (Beckman Coulter, cat # A63880) were used for RNA and DNA purification, respectively, at each required step. The pooled libraries were sequenced using MinION flow cells (R9.4.1) or Flongle cells with MinKNOW v21.06.0 (ONT). Raw reads were base-called, filtered (minimum Q score >7), debarcoded, and aligned with the *S.* Typhi ISP2825 genome^68^ or *S.* Typhimurium LT2 genome using Guppy v5.0.11 (ONT, RRID:SCR_023196). Normalization was performed with DESeq2 (alpha = 0.01, a cutoff for read counts = 10), and data were visualized with Prism v10 (GraphPad, RRID:SCR_002798). Genes with an adjusted *p* value (Padj) < 0.01 and log2 fold change >1 were considered statistically significant.

### Semi-qRT-PCR

Semi-qRT-PCR was performed as previously described.^69^ Briefly, total RNA (2 μg) was converted into cDNA using Maxima H minus RTase (Thermo Scientific, cat# EP0752) and RNaseOUT (Invitrogen, cat# 10777019). 20-fold diluted cDNA was subjected to qPCR amplification using a Green Taq polymerase (GenScript, cat #E00043) with gene-specific primers (Table S4). Gene expression levels were normalized to *rrsA* expression by quantifying amplicon band intensity using ImageJ (NIH, SCR_003070). For Figure S6, *S.* Typhi and mutant strains were cultured overnight in Miller LB medium. The seed culture (40 μL) was inoculated into 2 mL of LB containing 0.3 M NaCl and 10 mM potassium phosphate buffer (pH 6.7), with and without 0.2% GalNAc. Bacteria grew to an OD600nm of 1 (~3 h). Bacteria were collected, and total RNA was extracted using the RNAprotect Bacteria Kit (Qiagen cat#76506) and the RNeasy Mini Kit (Qiagen cat#74104) according to the manufacturer’s instructions. Complementary DNA was synthesized using the iScript cDNA Synthesis Kit (Bio-Rad Cat#1708891). Gene expression was analyzed by PCR, with 16S rRNA serving as the positive control. Additional details are provided in Table S6.

### Protein structure prediction

To identify *S.* Typhi outer membrane surface proteins (OMSPs) that recognize MUC2 and GalNAc differently from *S.* Tm, we screened candidates from functional *S.* Ty OMSPs based on the outer membrane protein database of *S.* Tm LT2. We analyzed the structures of 72 functional OMSPs from the AlphaFold database. We filtered candidates whose amino acid sequences in the extracellular (EC) domain differed from those of their *S.* Tm orthologs (17 proteins) (Table S2). The predicted *S.* Ty OmpW structure (UniProt: Q8Z7E2) was obtained from the AlphaFold Protein Structure Database (https://alphafold.ebi.ac.uk/) and compared with the *E. coli* OmpW crystal structure (PDB : 2F1T) to predict the topology of *S.* Ty OmpW amino acids relative to the outer membrane. All structures were analyzed and visualized using Chimera ×1.8. Protein homology detection of RS12510 was performed using the MPI Bioinformatics Toolkit HHpred (https://toolkit.tuebingen.mpg.de/tools/hhpred).^70^

### Transposon-directed insertion site sequencing

The plasmid pCat-mariner was transferred into *S.* Typhi P_*hilD*_-LacZ via conjugation, yielding approximately 50,000 colonies, representing 10-fold coverage of 4,688 *S.* Typhi genes. The library was grown in LB.3 medium with 0.2% MUC2 for 1 h, then plated on LB.3 agar containing X-gal (80 μg/mL) and 0.05% GalNAc. A total of 145 white colonies from three independent biological replicates were collected. These colonies were pooled and cultured in LB medium until an optical density at 600 nm (OD_600_) of approximately 1.0 was reached. Two milliliters of this culture were used for gDNA extraction with the QIAamp DNA Blood Mini Kit (Qiagen, cat. #51106). Three micrograms of gDNA were prepared with Ultra II End-prep mix (NEB, cat #E7546L), and a sequencing library was generated using the Nanopore Ligation Sequencing Kit (SQK-LSK114). Sequencing was performed with MinION flow cells (R10.4.1) using MinKNOW v21.06.0 (ONT), with base-calling done by Guppy v6.4.2 (minimum Q score >7). Reads containing the transposon sequence, AGACCGGGGACTTATCATCCAACCTGTTA, were isolated using seqkit amplicon and trimmed to 100 bp after the transposon. We identified 28 candidates (peak coverage >1), including the global transcription factor Fis (Table S3). Among these, we focused on genes with transposon insertion peak coverage >2 whose protein products include a periplasmic domain, as potential key players in signal transduction from the bacterial surface.

### β-galactosidase assay

*S.* Typhi strains carrying *E. coli* lacZ, transcriptionally fused with *S.* Typhi *hilD* promoter, were grown in LB.3 supplemented with or without 0.2% of each glycan or monosaccharide mix for 3 h. Cultured cells (80 μL) were diluted 10-fold in 0.06 M Na_2_HPO_4_, 0.04 M NaH_2_PO_4_, 0.01 M KCl, 1 mM MgSO_4_, and 0.04 M β-mercaptoethanol, and lysed with 20 μL of 0.1% SDS and 40 μL of chloroform at 37°C for 10 min. After adding 160 μL of ONPG (4 mg/mL), the β-galactosidase activity was measured as previously described.^71^

### *Ex vivo* epithelial cell invasion assay

Mice were euthanized using CO_2_, and the entire small intestines (from the end of the stomach to the terminal ileum) were collected. The intestines were washed with PBS to remove residual digesta. They were then immediately transferred to a Petri dish containing 5 mL of freshly prepared chelating buffer (54.9 mM D-sorbitol, 43.4 mM sucrose, and 5 mM EDTA in PBS).^72^ The tissues were minced into 1–2 mm^3^ pieces, and the suspension was incubated at room temperature for 10 min. After vortexing for 30 s, the cell suspensions were passed through a 70-μm cell strainer and then centrifuged at 500 × *g* for 10 min. The cell pellets were resuspended in 1 mL of DMEM containing Kanamycin, and 2 × 10^5^ cells were collected in an Eppendorf tube. The cells were then infected with *Salmonella* at an MOI of 30 for 1 h. Washing with PBS and gentamicin protection were performed as described in the invasion assays, using 24-well plates, except that the buffer removal step was replaced with centrifugation.

### Animal experiments

Littermates of males and females aged 6 to 12 weeks were used. Mice were fasted for 4 h before pre-treatment with 100 μL of 10% sodium bicarbonate, followed by infection via oral gavage with 100 μL of PBS containing 5 × 10^9^ CFU of *S.* Typhi or *S.* Typhimurium carrying the pWSK-mCherry plasmid. When indicated, Cmah null mice were pretreated with 100 μL of PBS containing 20 mg of streptomycin. After 48 h, mice were orally gavaged with 5 × 10^9^ CFU of *S.* Typhi or *S.* Typhimurium carrying pWSK-mCherry. To test the effects of MUC2 glycan components on *S.* Typhi infectivity, Muc2^−/−^ or streptomycin-pretreated Cmah−/− mice were orally gavaged with 100 μL of PBS containing 5 × 10^9^ CFU of *S.* Typhi ±300 mM monosaccharide mixtures. *S.* Typhi inoculum was cultured in the presence of either mix1 or mix4. At indicated times, infected mice were euthanized, and the organs (livers and spleens) were collected for CFU assays. In three-day infection studies (Figures 5B and 5D), mice were orally gavaged with 5 × 10^9^ CFU of *S.* Typhi or 5 × 10^6^ CFU of *S.* Typhimurium. When indicated (Figure 5C), Cmah null mice were infected intraperitoneally with 2 × 10^5^ CFU of *S.* Typhi *tviE* P263S, *S.* Typhi *tviE* P263S *ompW*_S.Tm_, or *S.* Typhimurium SL1344. The collected organs were mechanically homogenized with 5 mL of PBS containing 0.05% sodium deoxycholate. To prevent cross-contamination between samples, the homogenizer probe was washed three times with 100% EtOH and PBS. CFUs were determined by plating 10-fold serial dilutions of homogenates onto LB agar plates supplemented with kanamycin. The total CFU in each organ was calculated by multiplying the dilution factors.

## QUANTIFICATION AND STATISTICAL ANALYSIS

Statistical analyses were performed with GraphPad Prism 10 (GraphPad Software, RRID:SCR_002798). Bacterial RNA-seq normalization was performed with DESeq2, and semi-qRT-PCR band densitometry was performed with ImageJ (NIH, RRID:SCR_003070). For every experiment, the statistical test used, the exact value of *n*, what *n* represents, and the measures of center and dispersion are also specified in the corresponding figure legend.

For *in vitro* CFU, swarming, and qRT-PCR experiments (Figures 1, 2, 3, and, 4A, and related supplementary figures), data are presented as box-and-whisker plots showing all individual data points, with the box representing the 25th to 75th percentiles, the central line indicating the median, whiskers extending from the lowest to the highest values, and the mean and standard deviation (SD) of three to five independent biological replicates indicated. Unless otherwise specified, *n* refers to the number of independent biological replicates. For image-based quantification of intracellular STy and *S.* Typhimurium (Figures 1C and 1F), each data point represents the normalized bacterial count from a single image, and *n* = 120 images per condition were collected from three independent biological experiments. Two-tailed unpaired Student’s *t*-tests were used to compare the untreated control (UT) with each treatment group unless otherwise specified. For bacterial RNA-sequencing analysis (Figures 1I-1N; Table S1), reads were normalized with DE-Seq2 (alpha = 0.01; read-count cutoff = 10), and genes with an adjusted *p* value (Padj) < 0.01 and log_2_ fold change >1 were considered significantly differentially expressed.

For animal experiments (Figures 4C-4F, 4H-4I, and 5), *n* represents the number of individual mice, with each data point corresponding to one animal; both male and female age-matched littermates aged 6 to 12 weeks were used, with sex indicated in the figures (black dots, male; green dots, female). Mice were assigned to experimental groups such that each arm contained littermates of both sexes where available, in order to balance litter and sex across arms. Data are presented as box-and-whisker plots as described above. In Figure 4, two-tailed paired *t*-tests were used to compare intestinal segments (duodenum, jejunum, and ileum) from the same animal (Figures 4C-4F and 4H-4I). For the organ CFU data in Figure 5, two-tailed Mann-Whitney *U* tests were used between the indicated groups because organ CFU distributions were not assumed to follow a normal distribution.

Statistical significance is denoted as *p < 0.05, **p < 0.01, ***p < 0.001, ****p < 0.0001; n.s., not significant. Sample sizes for both *in vitro* and *in vivo* experiments were selected based on our laboratory’s prior experience with similar assays and on published studies using comparable STy invasion and murine oral gavage models, and were sufficient to detect the effect sizes reported. No formal *a priori* power calculation was performed. No data were excluded from any analysis; all biological and technical replicates that met the pre-established quality criteria (bacterial cultures reaching the target OD_600_, mice surviving to the assigned experimental endpoint, and images in which DAPI-stained nuclei were countable) were included. Investigators were not blinded during data collection or analysis; however, quantification relied on objective, largely automated readouts (colony counts on LB agar plates, the BZ-X810 image analyzer, DESeq2, and ImageJ densitometry) to minimize observer bias. For parametric tests (unpaired and paired *t*-tests), data were assumed to be approximately normally distributed based on the underlying assay type and inspection of the data distribution; where this assumption was less appropriate (organ CFU data with a broader dynamic range in Figure 5), non-parametric Mann-Whitney *U* tests were used instead.

## Supplementary Material

1

2

3

4

Supplemental information can be found online at https://doi.org/10.1016/j.celrep.2026.117801.

## Figures and Tables

**Figure 1.**
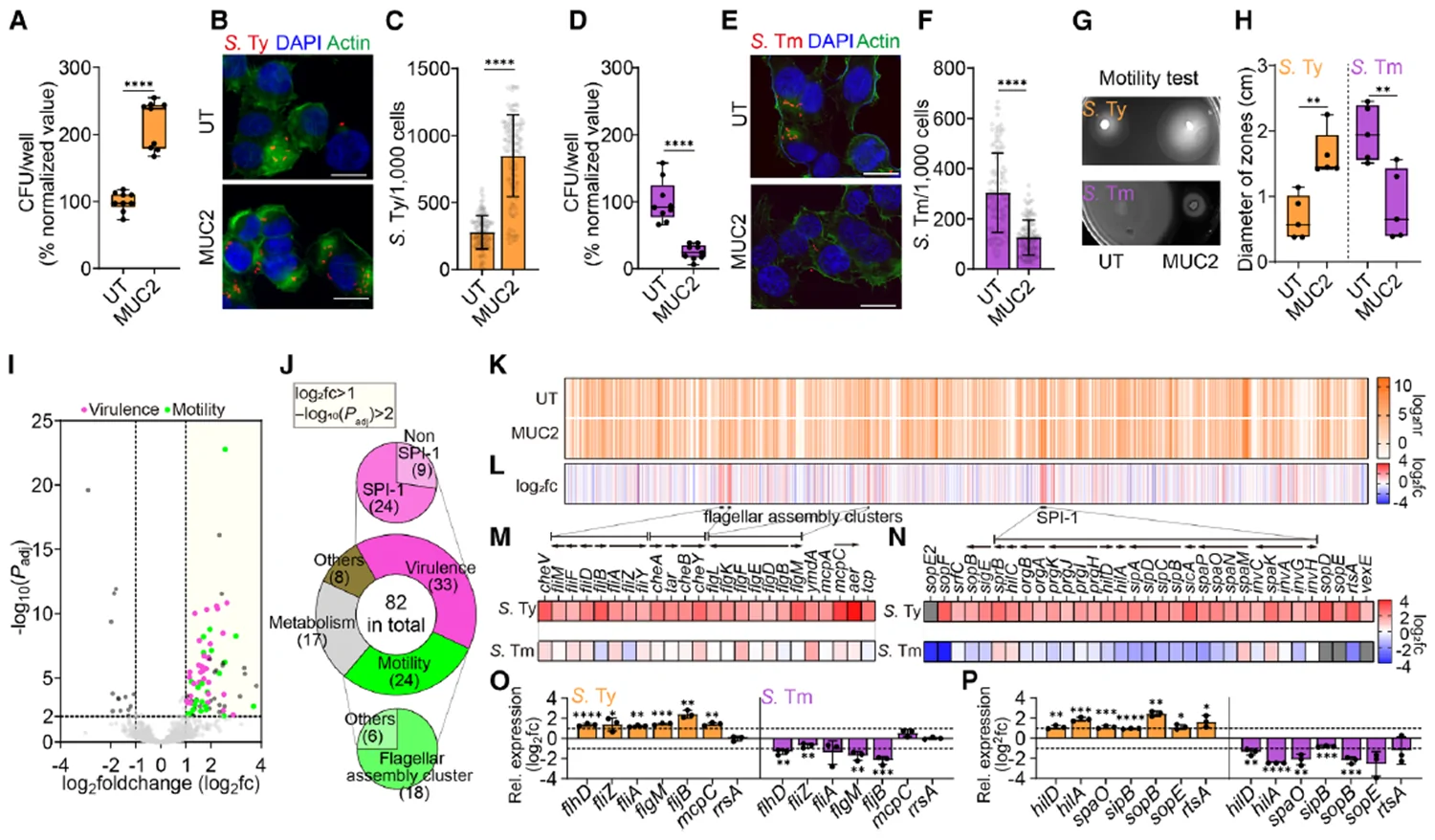
MUC2 is a cue promoting STy infectivity (A) CFU analyses of intracellular STy recovered from infected human epithelial cells (Henle-407) at 2 h post-infection. UT, STy cultured without MUC2. MUC2, STy cultured in LB.3 with 0.2% MUC2. (B) Representative fluorescence microscopy images of Henle-407 cells infected with STy cultured with or without MUC2. Red, STy (anti-O9 antibody). Green, F-actin (phalloidin). Blue, nuclei (DAPI). Scale bars, 20 μm. (C) Quantification of intracellular STy from three independent experiments corresponding to (B). Phalloidin-stained Henle-407 cells were imaged with a BZ-X810 image analyzer, and bacterial counts were normalized to the number of DAPI-stained nuclei per image.^15^ Each dot represents the value from one image. *n* = 120. (D) CFU analyses of intracellular STm. (E) Representative fluorescence microscopy images of Henle-407 cells infected with STm cultured with or without MUC2. Red, STm (anti-O4 antibody). Green, F-actin (phalloidin). Blue, nuclei (DAPI). Scale bars, 20 μm. (F) Quantification of intracellular STm from three independent experiments corresponding to (E), performed as in (C). *n* = 120. (G) Swarming motility of STy or STm cultured in LB.3 ± MUC2. (H) Quantitative data from five independent experiments related to (G). (I) Volcano plot shows differentially expressed genes (DEGs) of STy cultured in LB.3 in the presence of MUC2 relative to LB.3 without MUC2. DEGs linked to virulence and motility/chemotaxis are highlighted in magenta and green, respectively (log_2_fc > 1.0, −logPadj>2). (J) Pie chart summarizes STy transcripts significantly increased upon exposure to MUC2. (K) Heatmaps show normalized reads (nr) of all STy transcripts. (L) Heatmap shows log_2_fc values. (M) Log_2_fc values of motility/chemotaxis genes of STy (top) and STm (bottom) cultured in LB.3 + 0.2% MUC2. (N) Log_2_fc values of SPI1 genes of STy and STm. Arrows indicate the operons. (O) qRT-PCR data show fold changes of representative motility genes in MUC2-exposed STy (orange) and STm (purple) relative to UT. (P) qRT-PCR data show fold changes of representative virulence genes in MUC2-exposed STy (orange) and STm (purple) relative to UT. The box-and-whisker plot displays all data points, with whiskers extending from the lowest to the highest values. The box depicts the interquartile range (25th to 75th percentiles), with the median value indicated by the central line. The mean and standard deviation (SD) of 3–5 independent measurements are presented. Two-tailed unpaired *t* tests between UT and the tested samples were performed unless specified otherwise. *p < 0.05, **p < 0.01, ***p < 0.001, and ****p < 0.0001. See also Figures S1 and S2, and Table S1.

**Figure 2.**
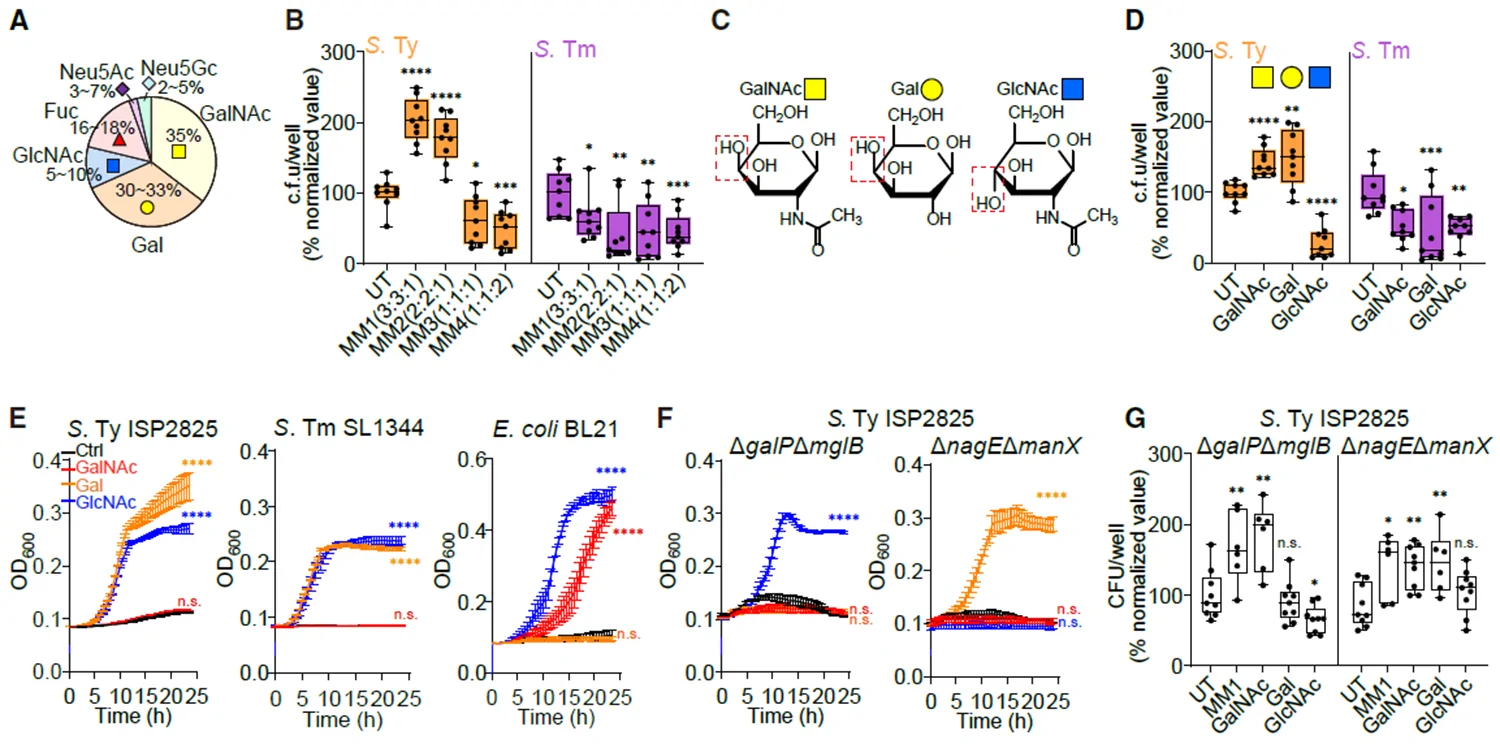
The main MUC2 glycan components are essential in enhancing *S.* Typhi infectivity (A) Pie chart shows MUC2 glycan components. (B) CFU analyses of intracellular *Salmonella* recovered from Henle-407 cells infected with STy and STm cultured in LB.3 containing the indicated monosaccharide mixtures (MM). (C) The pyranose ring structures of GalNAc, Gal, and GlcNAc, with chiral centers at carbon 4 highlighted. (D) CFU analyses of intracellular *Salmonella* recovered from Henle-407 cells infected with STy and STm cultured in LB.3 containing indicated glycans. (E) Growth curves of STy ISP2825, STm SL1344, and *E. coli* BL21 cultured in minimal media containing indicated glycans as the sole carbon source. Media without any carbon source were used as negative controls. (F) Growth curves of STy mutants lacking galactose *(ΔgalPΔmglB*) or GlcNAc *(ΔnagEΔmanX*) transporters. (G) CFU analyses of intracellular *Salmonella* recovered from Henle-407 cells infected with the indicated STy mutants that were cultured in a minimal medium supplemented with the indicated glycan. Box-and-whisker plots. Three or more independent experiments. Two-tailed unpaired *t* tests between UT and indicated samples were performed unless otherwise specified. *p < 0.05, **p < 0.01, ***p < 0.001, and ****p < 0.0001. See also Figures S3 and S4.

**Figure 3.**
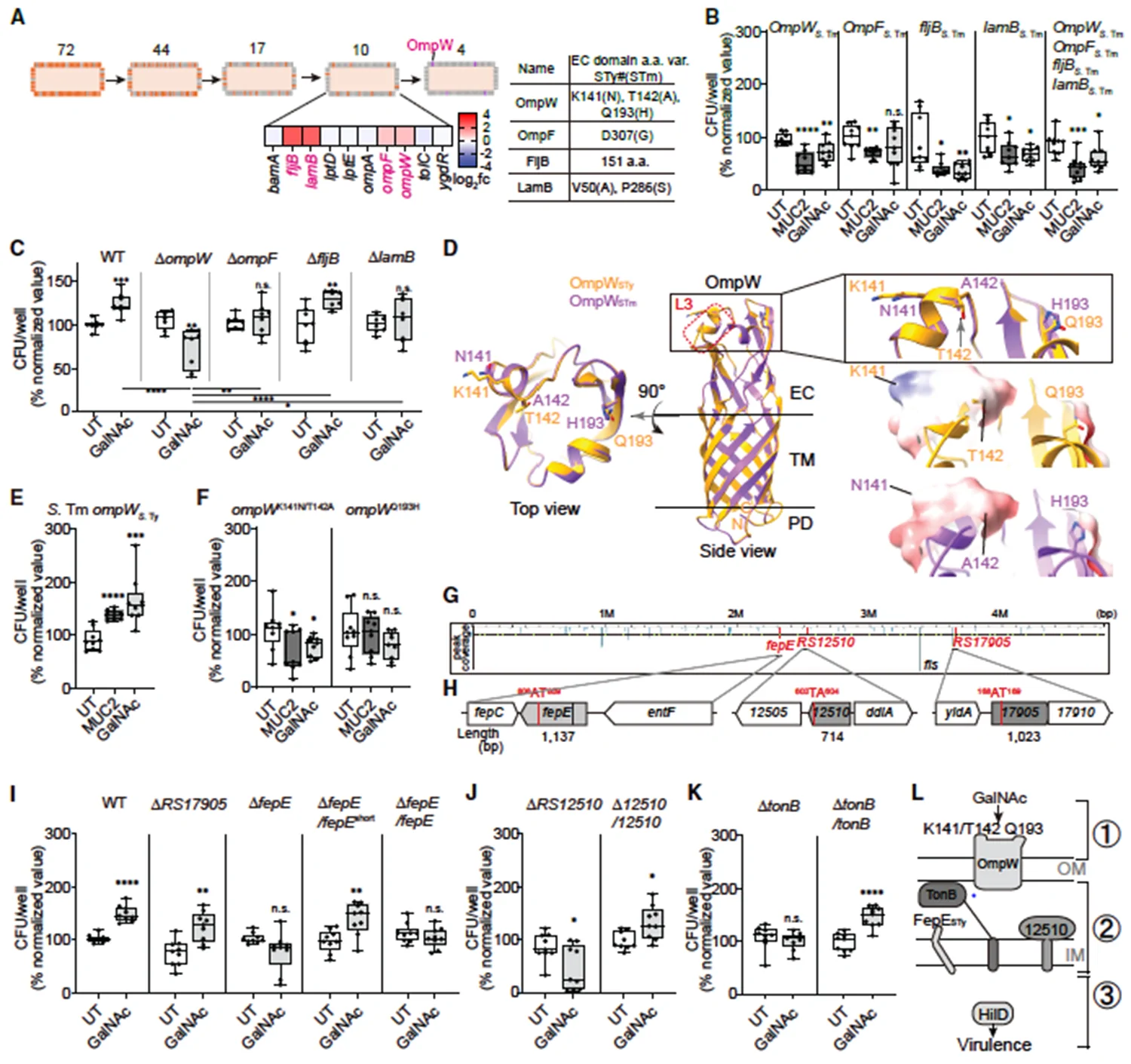
OmpW is a surface protein of *S.* Typhi, and the extracellular domain residues, K141, T142, and Q193, are crucial for the GalNAc-mediated enhanced infectivity (A) Schematic workflow shows STy outer membrane surface proteins (OMSPs) that are likely responsible for MUC2 glycan components-mediated enhanced infectivity. Heatmap, mRNA expression profiles for 10 OMSP candidates. Table, EC domain amino acid residue differences between STy and STm. (B) CFU analyses of STy chimera strains for the indicated OMSPs. Bacteria were cultured in buffered LB.3 containing MUC2 or GalNAc, with LB.3 (UT) serving as a negative control. (C) CFU analyses of STy deletion strains for the indicated OMSPs. Bacteria were cultured in buffered LB.3 with or without GalNAc. (D) Structural comparison of STy OmpW (orange) and STm OmpW (purple), predicted using AlphaFold. The orientation and membrane topology were determined based on the *E. coli* OmpW structure (PDB: 2F1T). Three amino acid residues that differ between STy and STm are highlighted in orange and purple, respectively. (E) CFU analyses of STm *ompW*_STy_ cultured in LB.3 containing MUC2 or GalNAc, with UT as a negative control. (F) CFU analyses of STy strains carrying *ompW* K141N/T142A or Q193H, cultured in LB.3 containing MUC2 or GalNAc, with UT as a negative control. (G) Transposon insertion sites across the STy genome. (H) Genomic loci of the candidate genes *fepE*, *RS12510*, and *RS17905*. (I) CFU analysis of WT, STy*ΔRS17905,* STy*ΔfepE,* and the in-trans-complemented strains cultured in LB.3 ± GalNAc. (J) CFU analysis of STy*ΔRS12510* and the in-trans-complemented strain cultured in LB.3 ± GalNAc. (K) CFU analysis of STy*ΔtonB* and the in-trans-complemented strain cultured in LB.3 ± GalNAc. (L) Schematic shows the GalNAc-OmpW-mediated mechanism. The extracellular ① and part of the cytoplasmic ③ mechanisms are revealed in this study. The peripheral domains of the identified bacterial determinants are essential for the periplasmic ② mechanism, while how they work together and how the signal is relayed to the downstream ③ mechanism remain unclear. Box-and-whisker plots. Three or more independent experiments. Two-tailed unpaired *t* tests between UT and the indicated samples were performed unless otherwise specified, with lines indicating the two specific groups. *p < 0.05, **p < 0.01, ***p < 0.001, and ****p < 0.0001. See also Figures S5-S10 and Tables S2 and S3.

**Figure 4.**
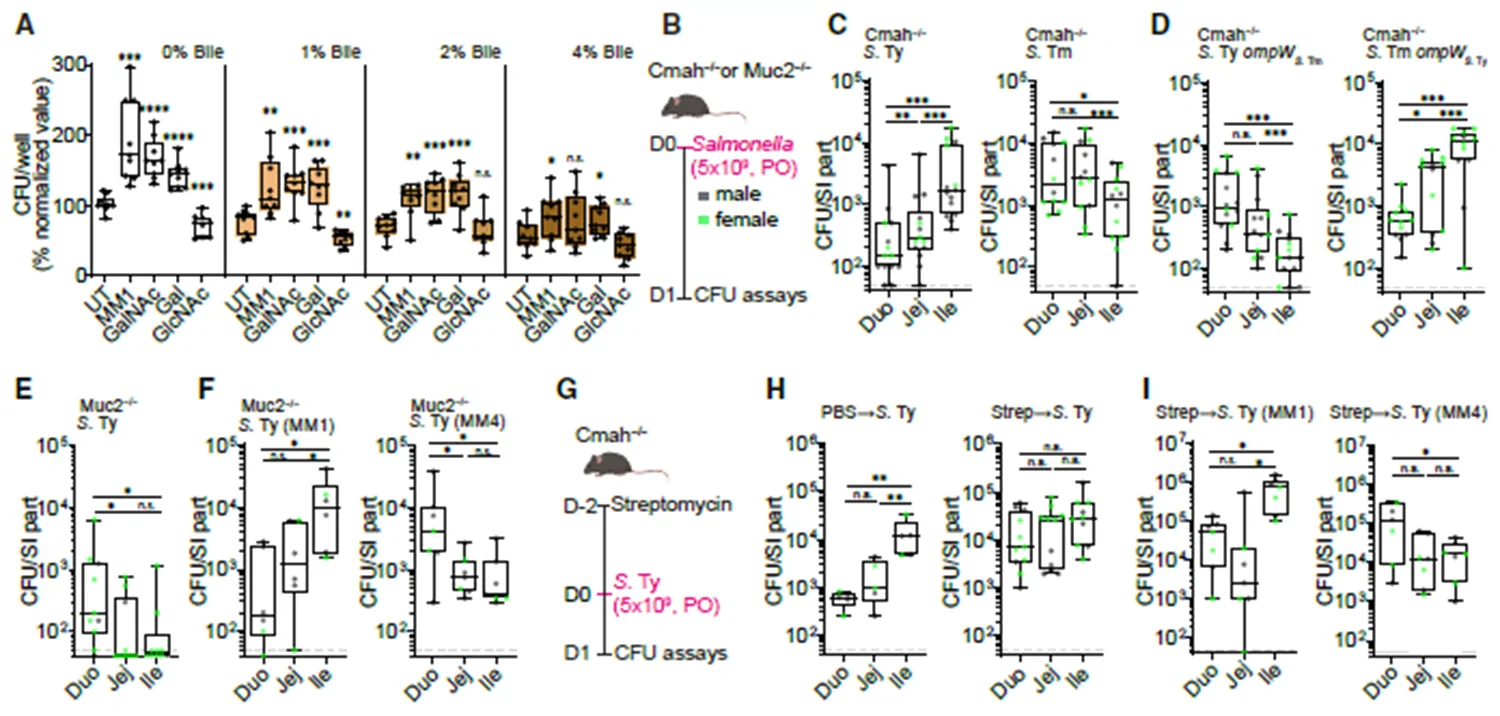
OmpW-mediated responses to MUC2 glycan components are linked to its ileum preference (A) CFU analysis of STy cultured in LB.3 in the absence or presence of 0.2% MM1 or indicated glycans, with varying bile acid concentrations (0, 1, 2, or 4%). (B) Schematic of animal studies associated with (C–F). PO, oral gavage infection. Black and green dots indicate data from male and female mice, respectively. (C) CFU data from Cmah null mice orally infected for 1 day with STy (left image) or STm (right image). Duo: duodenum; Jej: jejunum; Ile: ileum. (D) CFU data from Cmah null mice orally infected for 1 day with STy *ompW*_STm_ (left image) or STm *ompW*_STy_ (right image). (E) CFU data from Muc2 null mice orally infected for 1 day with STy. (F) CFU data from Muc2 null mice orally infected for 1 day with STy and orally administered MM1 (left image) or MM4 (right image). The inoculum was prepared by culturing in LB.3 containing MM1 or MM4 for 3 h. (G) Schematic of animal studies associated with (H-I). (H) CFU data from Cmah null mice pretreated with either PBS (left image) or streptomycin (right image), followed by oral infection for 1 day with STy. (I) CFU data from Cmah null mice pretreated with streptomycin, followed by oral infection for 1 day with STy, plus oral administration of MM1 (left image) or MM4 (right image). The inoculum was prepared by culturing in LB.3 containing MM1 or MM4 for 3 h. Box-and-whisker plots. Two-tailed unpaired *t* tests (a, between UT and treatment group) and two-tailed paired *t* tests (C-F, H-I) were performed between the indicated two groups. *p < 0.05, **p < 0.01, ***p < 0.001, and ****p < 0.0001; n.s., not significant. See also Figures S11 and S12.

**Figure 5.**
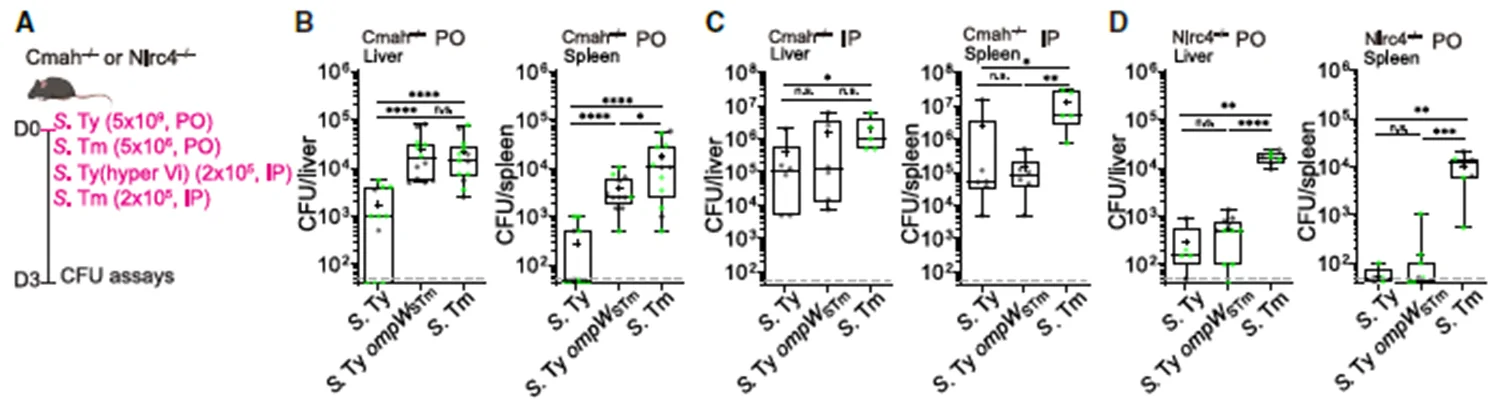
*S.* Typhi expresses OmpW_STm_ and serves as a promising model for studying oral STy infection (A) Schematic of animal studies associated with (B-D). (B) Organ CFU data from Cmah null mice orally infected (PO) for 3 days with STy, STy *ompW*_STm_, or STm. (C) Organ CFU data from Cmah null mice intraperitoneally (IP) infected for 3 days with STy, STy *ompW*_STm_, or STm. (D) Organ CFU data from Nlrc4 null mice orally infected for 3 days with STy, STy *ompW*_STm_, or STm. Box-and-whisker plots. Two-tailed Mann-Whitney *U* tests were performed between the indicated two groups. *p < 0.05, **p < 0.01, ***p < 0.001, and ****p < 0.0001; n.s., not significant. See also Figures S13 and S14.

**Table:** KEY RESOURCES TABLE

REAGENT or RESOURCE	SOURCE	IDENTIFIER
Chemicals, peptides, and recombinant protein		
5-bromo-4-chloro-3-indolyl-β-D-galactopyranoside	Goldbio	Cat# 7240-90-6
Galactose	Sigma	Cat# 59-23-4
*N*-acetylgalactosamine (GalNAc)	Cayman	Cat# 1811-31-0
*N*-acetylglucosamine (GlcNAc)	Sigma	Cat# 7512-17-6
Gentamicin	Lonza	Cat# 7-8518Z
Lysozyme	Sigma	Cat# L6876
Pierce Protease inhibitor tablet, EDTA-free	Thermo Fisher Scientific	Cat# A32965
Antibodies		
Rabbit anti-O9 antibody	SSIDiagnostica	REF40276; RRID: AB_2934032
Rabbit anti-O4 antibody	Hardy Diagnostics	M1031
Bacterial strains		
*S.* Typhi ISP2825	Table S5	Table S5
*S.* Typhimurium SL1344	Table S5	Table S5
Critical commercial assays		
RNeasy mini kit	Qiagen	74104
RNAprotect bacteria kit	Qiagen	76506
RNase-Free DNase set	Qiagen	79254
Qubit RNA HS Assay kit	Invitrogen	Q32852
Thermostable RNase H	NEB	M0523S
DNase I-XT	NEB	M0570S
Maxima H Minus Double-Stranded cDNA synthesis kit	Thermo Fisher	K2561
Ultra II End-prep mix	NEB	E7546L
Native Barcoding Expansion	ONT	EXP-NBD104687
Adaptor ligation kit	ONT	EXP-NBD104
RNAclean XP beads	Beckman Coulter	A63987
AMPure XP beads	Beckman Coulter	A63880
RNaseOUT	Invitrogen	10777019
Green Tag polymerase	Genscript	E00043
QIAamp DNA Blood Mini kit	Qiagen	51106
Nanopore Ligation Sequencing kit	ONT	SQK-LSK114
Bradford reagent	Bio-Rad	5000006
MinION flow cells	ONT	R9.4.1/R10.4.1
Flongle cells	ONT	Flongle
Deposited data		
RNA-seq data	GEO	GSE337684, GSE337685
TN-seq data	GEO	GSE337718
Experimental models: Cell lines		
Henle407	Originally obtained from ATCC and rederived	Validated using intestinal epithelial markers
Experimental models: Organisms/Strains		
Cmah null mouse (B6.129X1-Cmah ^tm1Avrk^/J)	Originally obtained from Jackson Lab	Bred at Cornell animal facility vivarium
Muc2 null mouse	Originally obtained from the Waldor Lab	Bred at Cornell animal facility vivarium Crossed with Cmah null mice
Nlrc4 null mouse	Originally obtained from the Vance Lab via Taconic	Bred at Cornell animal facility vivarium Crossed with Cmah null mice
Oligonucleotides		
Oligonucleotides	Integrated DNA Technologies (IDT)	Tables S4 and S6
Recombinant DNA		
Plasmids	Table S5	Table S5
Software and algorithms		
Alphafold2		https://alphafoldserver.com/
HHpred	MPI Bioinformatic Toolkit HHpred	https://toolkit.tuebingen.mpg.de/tools/hhpred
Oligo-ASST	M.T.Lee lab	https://oligo-asst.herokuapp.com/
DESeq2	DESeq2 Bioconductor	N/A
GraphPad Prism 10	GraphPad Software	https://www.graphpad.com/scientific-software/prism/
Guppy v.5.0.11/v6.4.2	ONT	https://nanoporetech.com/document/Guppy-protocol
MinKNOW v21.06.0	ONT	N/A
Excel	Microsoft	Office 365
Unicorn 6.3	GE Healthcare Life Science	https://www.cytivalifesciences.com/en/us/shop/unicorn-6-3-p-01118
ImageJ	National Institutes of Health	https://imagej.nih.gov/ij/
BZ-X810	KEYENCE	https://www.keyence.com
ChimeraX	UCSF ChimeraX	https://www.cgl.ucsf.edu/chimerax/
Image Lab	Bio-Rad	https://www.bio-rad.com
iBright Analysis	Thermo Fisher Scientific	https://www.thermofisher.com
Other		
96-well microplate	Falcon	353072
24 well	Corning	3526
DMEM high glucose	Corning	10-017-CV
DMEM	Corning	45000-312
FBS	Phoenix Scientific	PS-100
HBSS	Corning	21-020-CV
Plan Fluorite 20× LDPH objective	Keyence	BZ-PF20LP
Tecan Infinite M Plex	Tecan	RRID: SCR_026522

## Highlights

- *Salmonella* Typhi senses MUC2 glycans to drive invasion and ileal tropism
- Three extracellular residues of OmpW recognize N-acetylgalactosamine in MUC2
- A FepE-RS12510-TonB periplasmic relay links surface sensing to HilD activation
- An OmpW triple-mutant *Salmonella* Typhi strain enables a murine oral typhoid model
