# Supplementary material for: OmpW-mediated sensing of MUC2 GalNAc contributes to the ileal tropism of Salmonella Typhi
Source: Cell Rep. Author manuscript; Available in PMC 2026 Oct 6. (PMC13640848; doi:10.1016/j.celrep.2026.117801)
Supplement: Supplementary file 1 [file NIHMS2206914-supplement-1.pdf]

Cell Reports, Volume *45*

Supplemental information

OmpW-mediated sensing of MUC2 GalNAc contributes to the ileal tropism of *Salmonella* Typhi

Chang-Kyu Yoon, Gi Young Lee, Chanmi Kim, Katharina Ribbeck, and Jeongmin Song

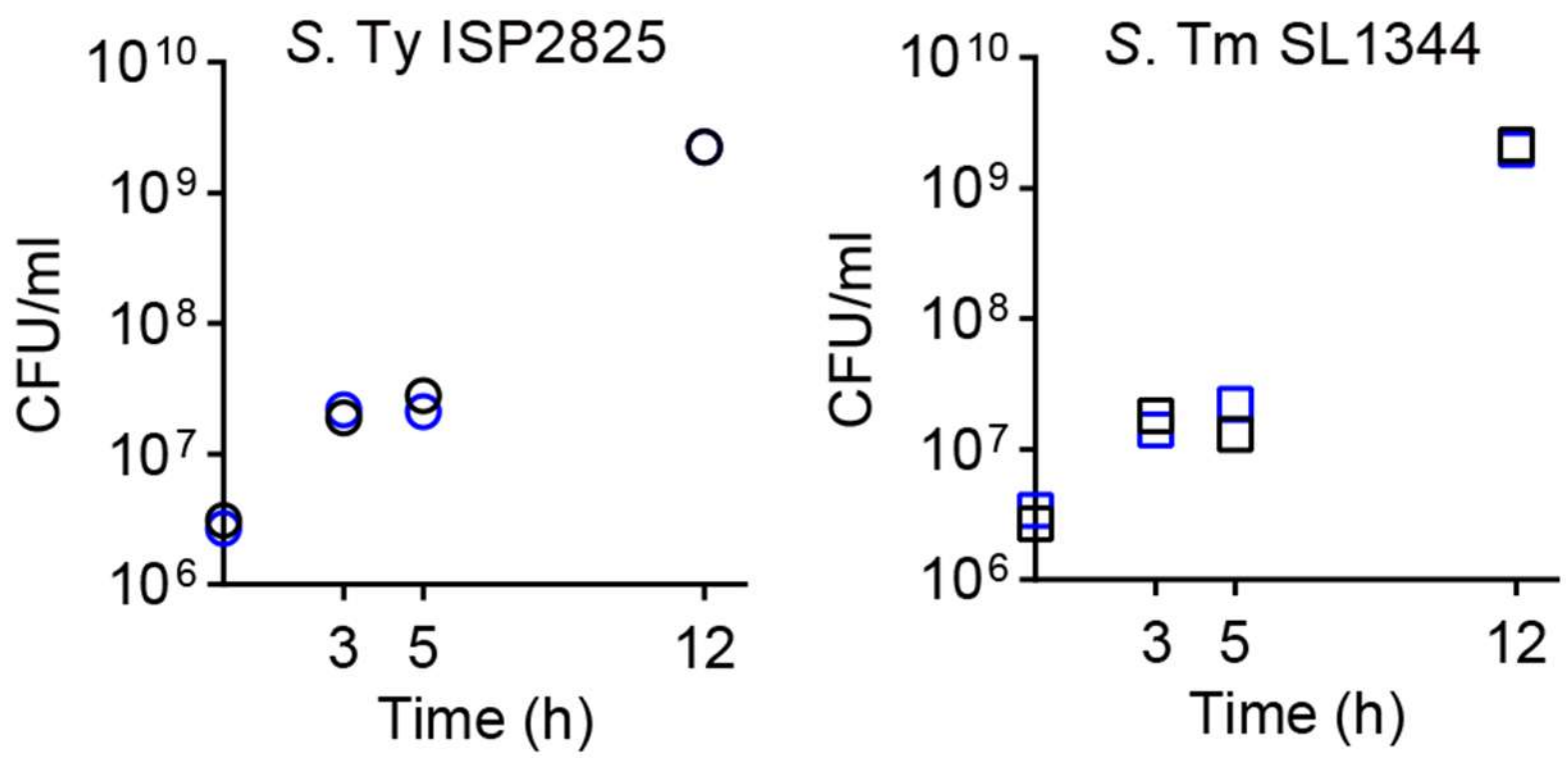

Figure S1, related to Fig. 1A-H. MUC2 does not influence *Salmonella* growth. *S.* Typhi ISP2825 or *S.* Typhimurium SL1344 were cultured in high-salt LB (LB.3) with or without purified porcine MUC2. The culture was serially diluted 10-fold at each time point, plated on LB.3 agar plates, and the number of CFUs was counted. These CFU counts were used to determine the total bacterial count in the culture. CFU data from cultures with or without MUC2 are shown with black and blue symbols, respectively.

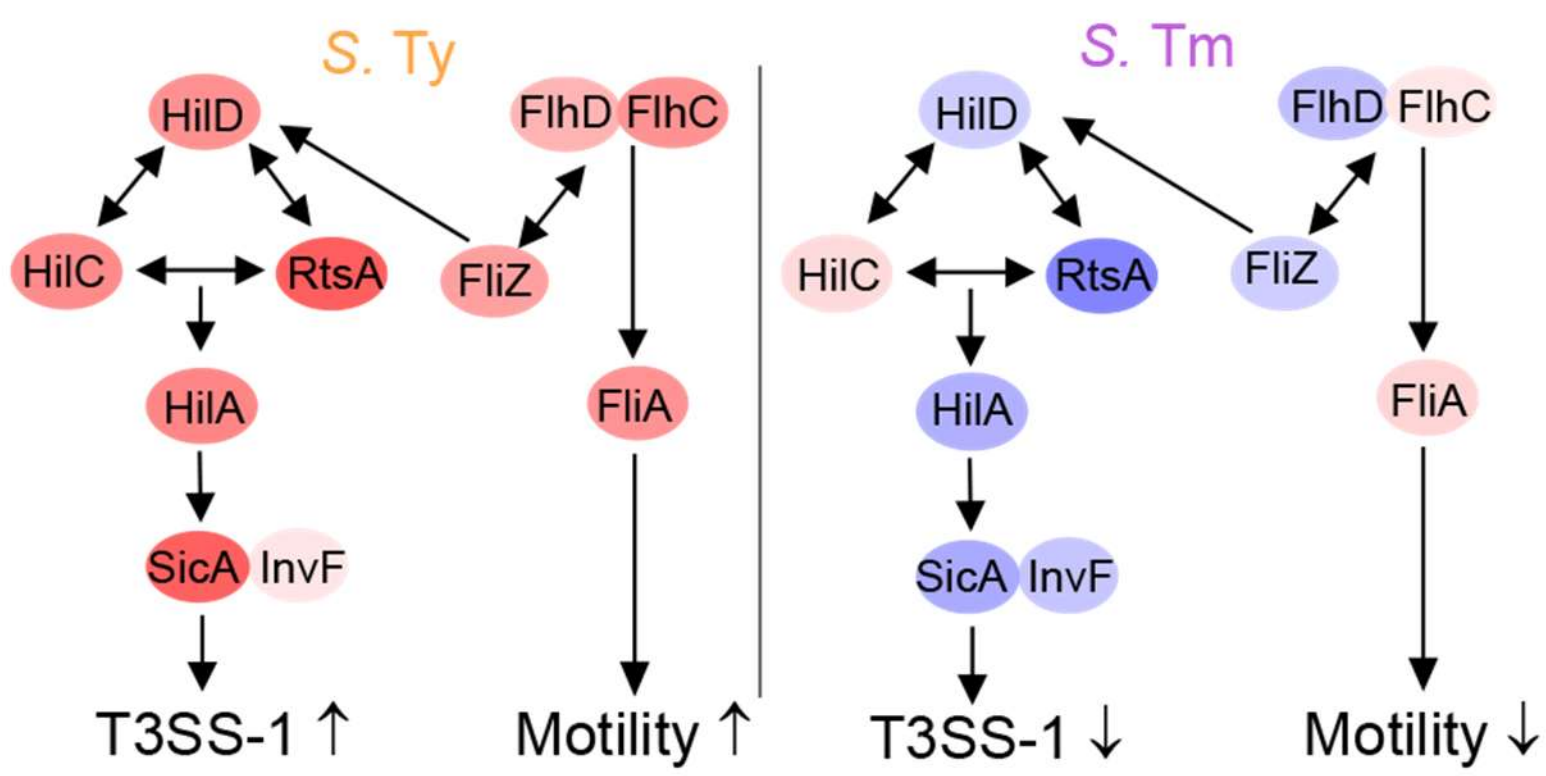

Figure S2, related to Fig. 1M and N. RNA sequencing results summarizing MUC2-mediated regulation of the transcriptional regulatory network of SPI-1 and motility genes in *S.* Typhi and *S.* Typhimurium. The expression changes of the indicated genes in the MUC2-treated *Salmonella* relative to untreated counterparts were determined by RNA sequencing and are summarized here.

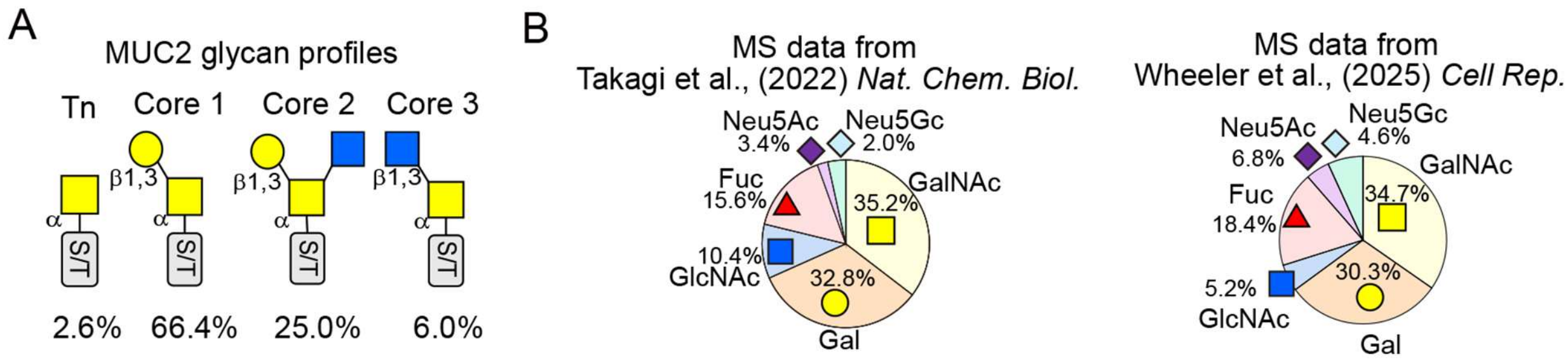

Figure S3, related to Fig. 2A. MUC2 glycan profile.

(A) The relative abundance of *O*-glycans from GalNAc-initiated core type structures in porcine MUC2. *N*-acetylgalactosamine (GalNAc), yellow square; Gal, yellow circle; *N*-acetylglucosamine (GlcNAc), blue square.

(B) The relative abundance of MUC2 glycan components is shown based on the MS data sets from the indicated published studies.

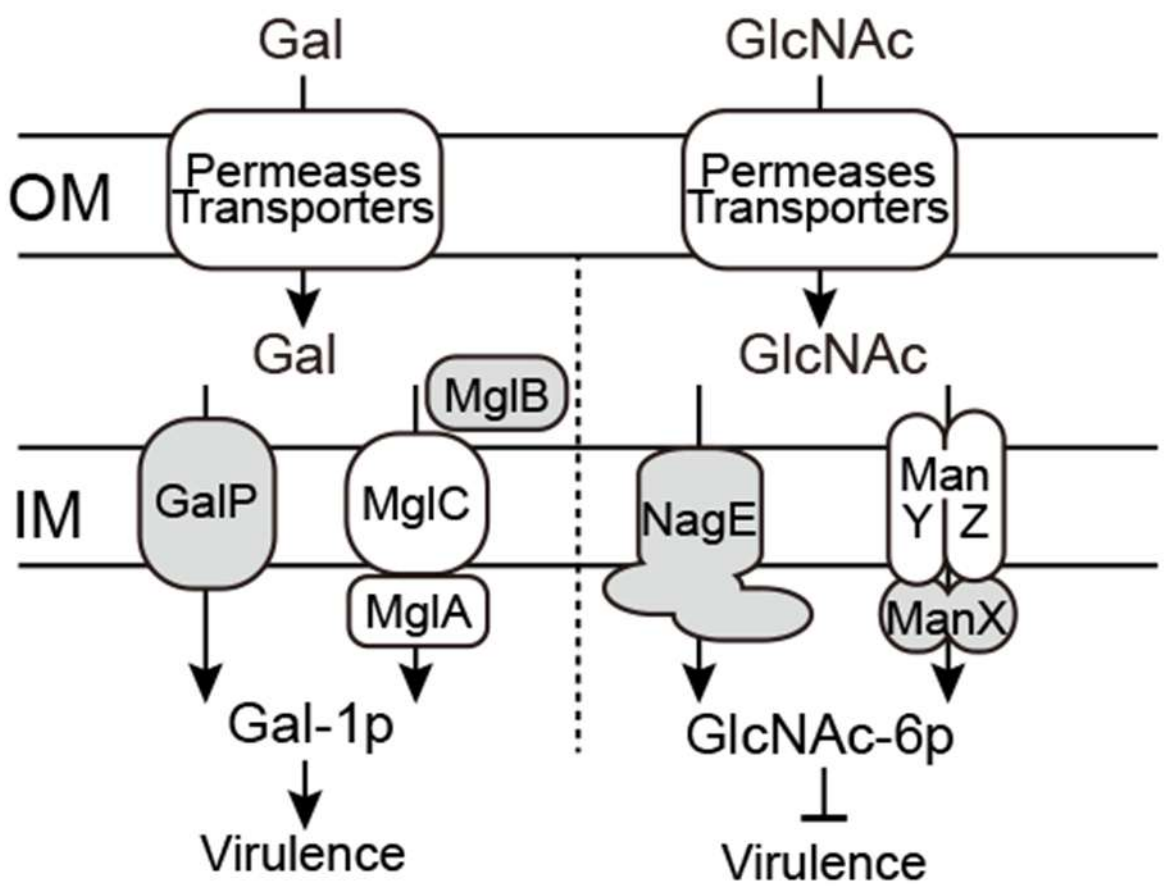

Figure S4, related to Fig. 2G. Galactose and GlcNAc-mediated signal transduction pathways in *S.* Typhi. The schematic overview of galactose and GlcNAc transport and signal transduction pathways connected to the transcriptional regulation of infectivity in *S.* Typhi. Galactose and GlcNAc are transported into the cell via outer membrane permeases and transporters. Specifically, galactose is transported into the cell by the inner membrane facilitative transporter GalP and the ABC transporter MglABC. GlcNAc is mainly taken up through the GlcNAc-specific phosphoenolpyruvate (PEP):carbohydrate phosphotransferase system (PTS) (NagE) and mannose PTS (ManXYZ). OM, outer membrane; IM, inner membrane.

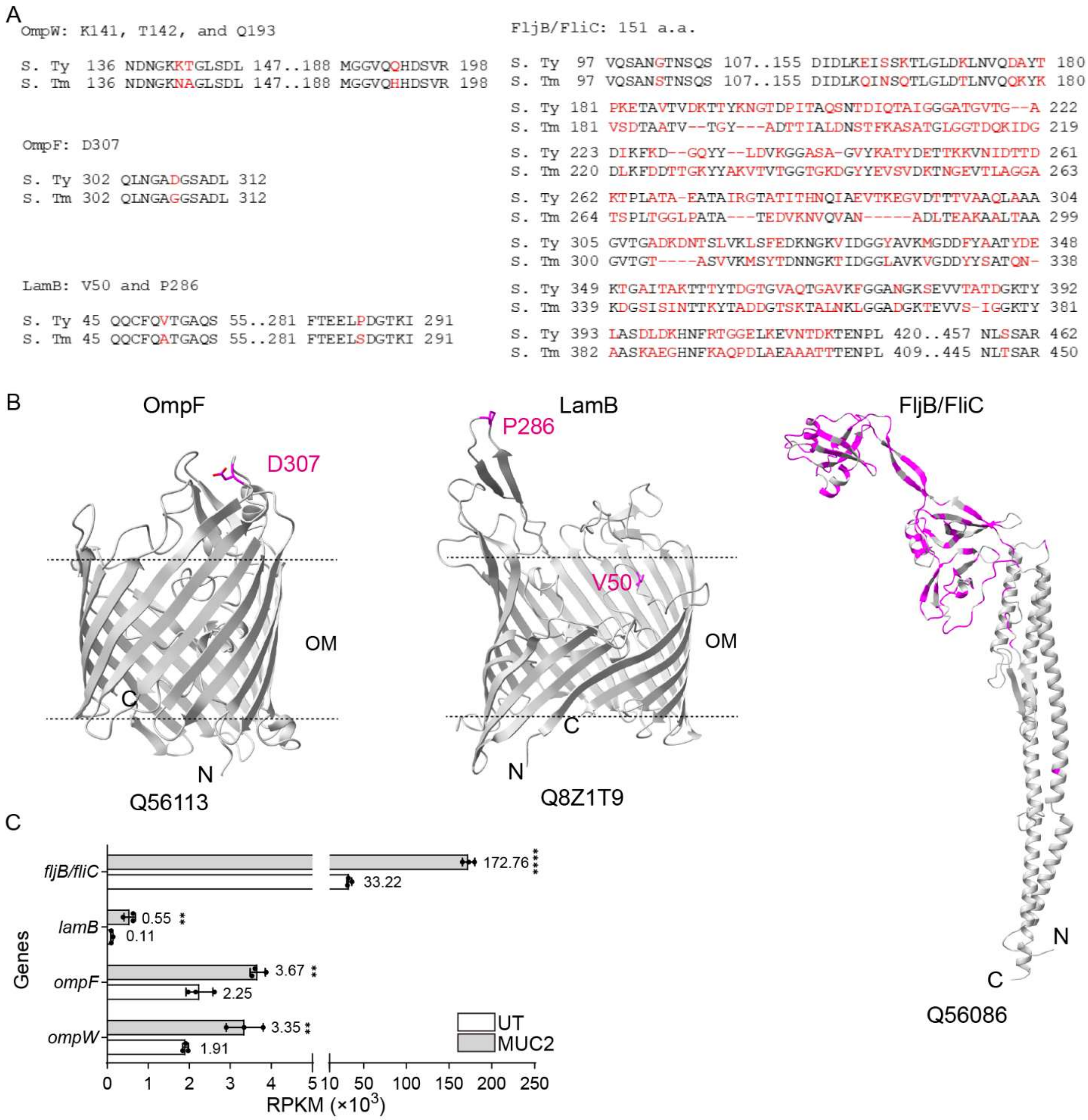

Figure S5, related to Fig. 3A-C. OmpW, OmpF, LamB, and FljB flagellin proteins.

(A) Comparative amino acid sequence alignments of OmpW, OmpF, LamB, and FljB between *S.* Typhi ISP2825 and *S.* Typhimurium SL1344. The variations in amino acid residues in the extracellular domain are indicated with red letters.

(B) AlphaFold-predicted structures of *S.* Typhi OmpF, LamB, and FljB flagellin. The amino acid residues different from those in *S.* Typhimurium are marked in magenta. UniProt entry codes are indicated below the structures. The N-termini and C-termini are labeled with 'N' and 'C', respectively.

(C) The RPKM values of *ompW, ompF, lamB,* and *fljB* are displayed.

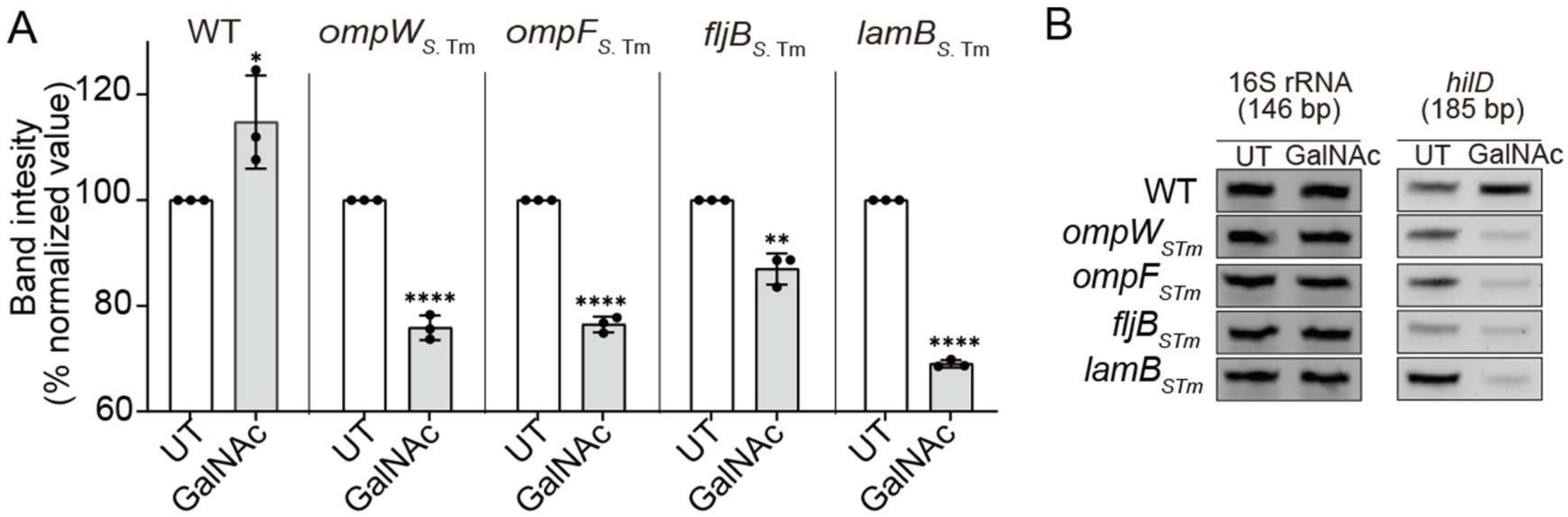

Figure S6, related to Fig. 3B. The effect of GalNAc on *hilD* gene expression in each STy ORF gene-swap mutant.

(A) Semi-qRT-PCR results of WT STy and the indicated chimera strains left untreated or treated with GalNAc. STy strains were cultured in buffered LB.3 in the presence and absence of GalNAc. qPCR conditions are listed in Table S6. Densitometric analysis was performed using ImageJ. 16s rRNA was used as a loading control. Bars represent the mean ± standard deviation (SD). 3 experiments. Each dot represents one experiment. Two–tailed t-tests were performed between the UT and GalNAc samples. *P<0.05; **P < 0.01; ****P < 0.0001.

(B) Representative gel images. See Table S6 for reaction conditions.

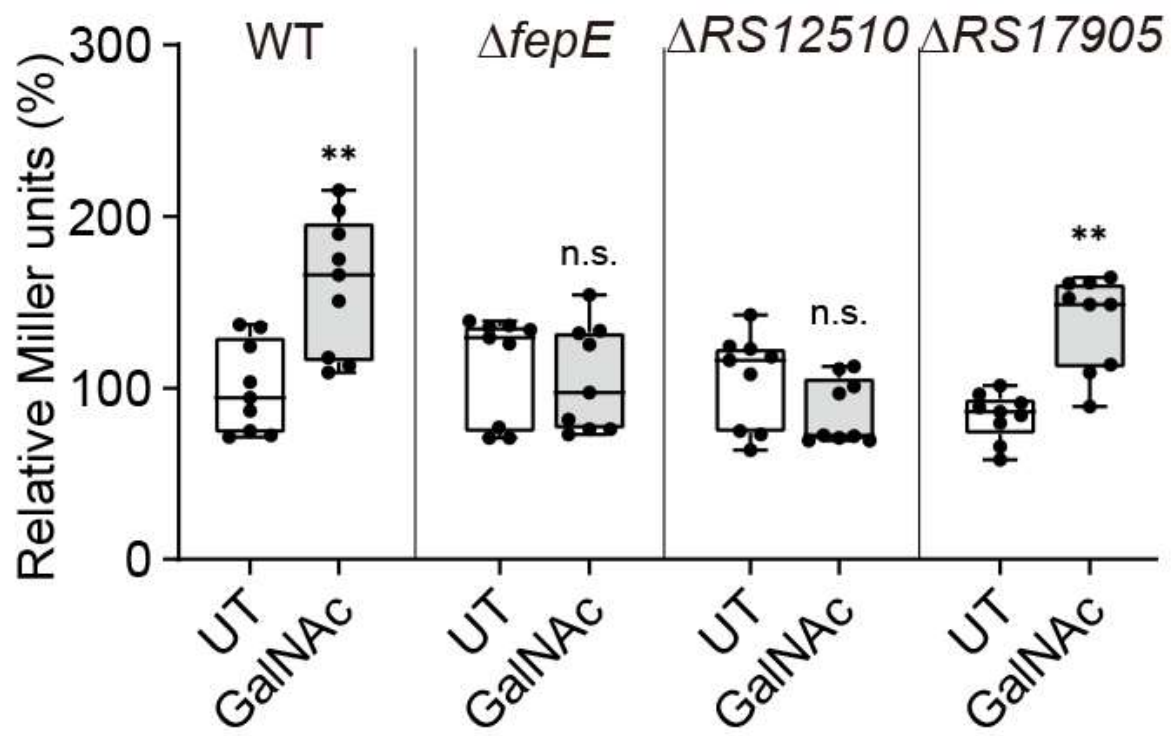

Figure S7, related to Fig. 3G-H. The effect of GalNAc on *hilD* promoter activity in each STy periplasmic partner ORF-deletion mutant.
The *hilD* promoter assay. WT STy and the indicated ORF-deletion mutants were cultured in buffered LB.3 with and without GalNAc. Each dot represents one biological replicate. Three experiments. Two–tailed unpaired t-tests were performed between UT and GalNAc samples. **$P < 0.01$; n.s., not significant.

Note that while this promoter assay method is effective at detecting increases in *hilD* promoter activity, it lacks the sensitivity to detect small decreases in activity.

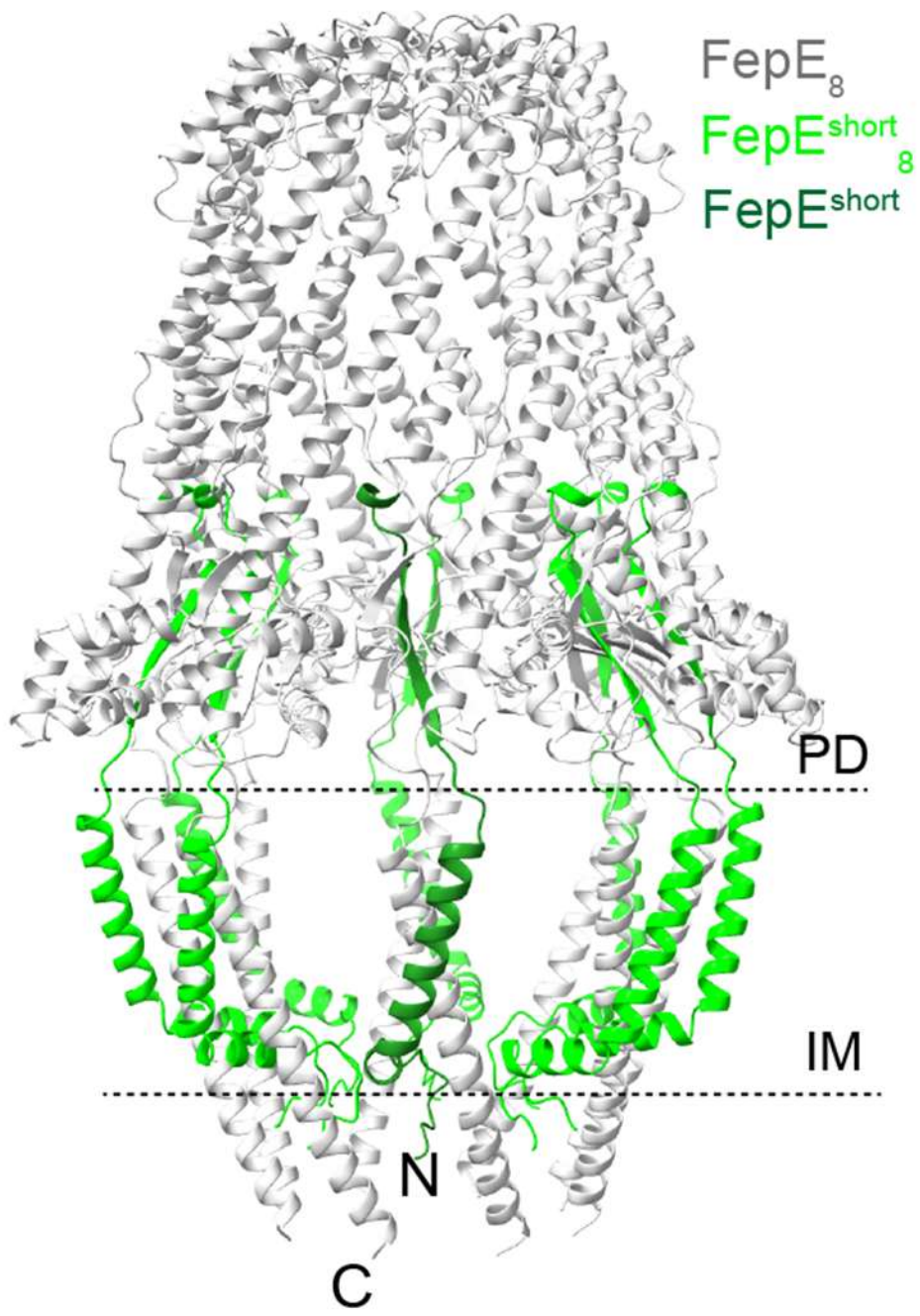

MPSLNVKQEKNQSFAGYSLPPANSHEIDLFSLIEVLWQAKRRILATVFAFACVGLLLSFL
LPQKWTSQAIVTPAESVQ*QGLERTLTALRVLDMEVSVDRASVFNLFIKKFSSPSLLEEY
LRSSPYVMDQLKGAQIDEQDLHRAIVVLSEKMKAVDSNAGKKNETSLFTSWTLSFTAPTR
EEAQKVLAGYIQYISDIVVKETLENIRNQLEIKTRYEQEKLAMDRVRLKNQLDANIQRLH
YSLEIANAAGIKRPVYSNGQAVKDDPDFSISLGADGISRKLEIEKGVTDVAEIDGDLRNR
QYYVEQLAAMNVSDVKFTPFKYQLSPSLPVKKDGPGKAIIIILAALIGGMMACGGVLLRH
AMVSRKMENALAIDERLV

Figure S8, related to Fig. 3I and L. Structural analysis of *S.* Typhi FepE (FepE_STy or FepE^{short}).
(Top) Given that WzzB assembles into an octameric complex with bell-shaped structures with hollow interior cavities, we predict that *S.* Typhi FepE^{short} (green) is also assembled as an octamer. Gray, the AlphaFold-predicted octamer structure of full-length FepE (FepE$_8$). Green, FepE^{short} octamer. Dark green, FepE^{short} monomer.
(Bottom) *S.* Typhi FepE amino acid sequence.

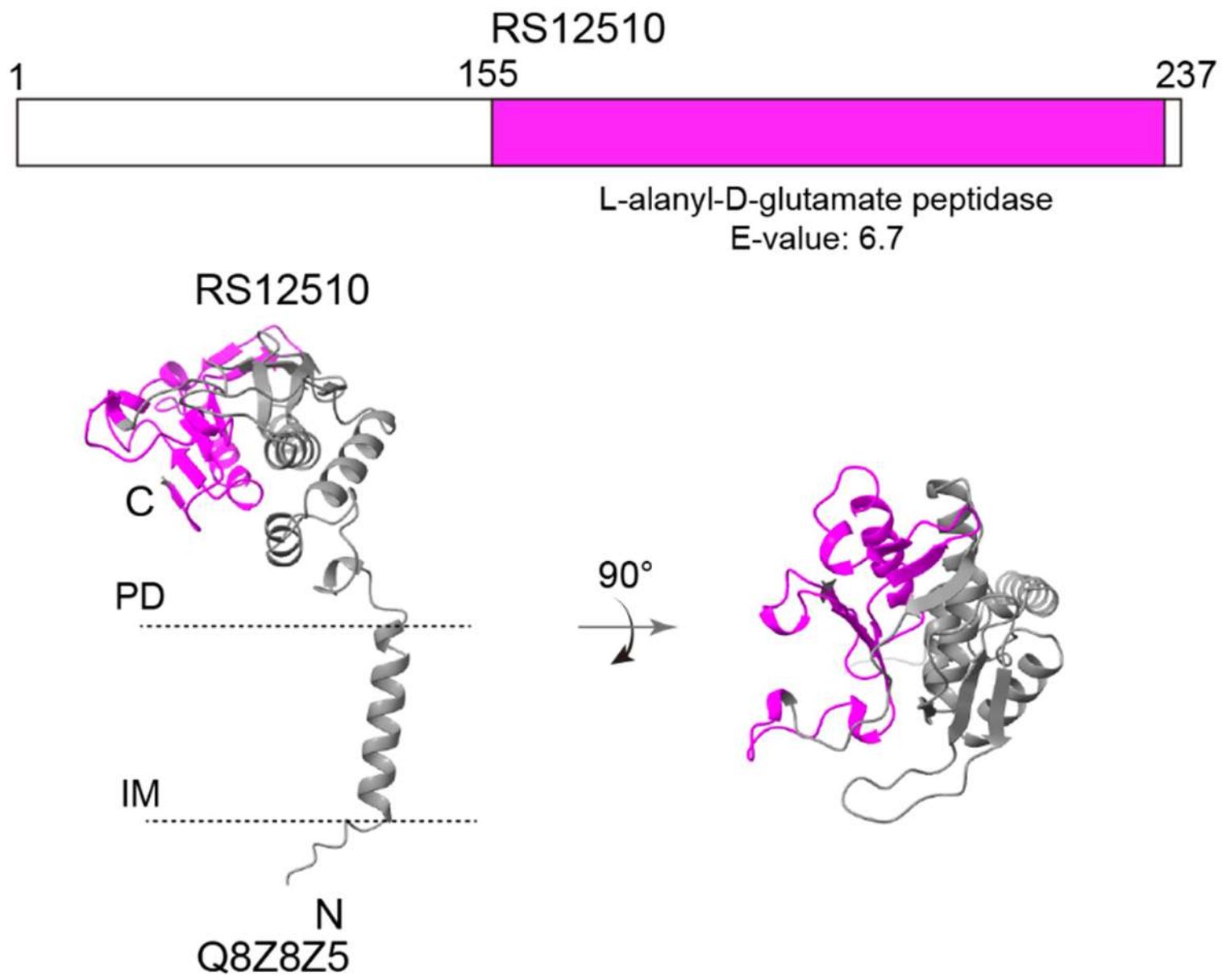

Figure S9, related to Fig. 3J and L. Structural analysis of RS12510.
(Top) The line diagram of the full-length RS12510. HHpred analysis predicted that the C-terminal region (a.a. 155−237) is homologous to the enzymatic domain of L-alanyl-D-glutamate peptidase.
(Bottom) AlphaFold-predicted full-length RS12510.
We note that Foldseek predicts that RS12510 matches a domain found in extensin-family structural proteins. Further investigation is warranted to define the role of RS12510.

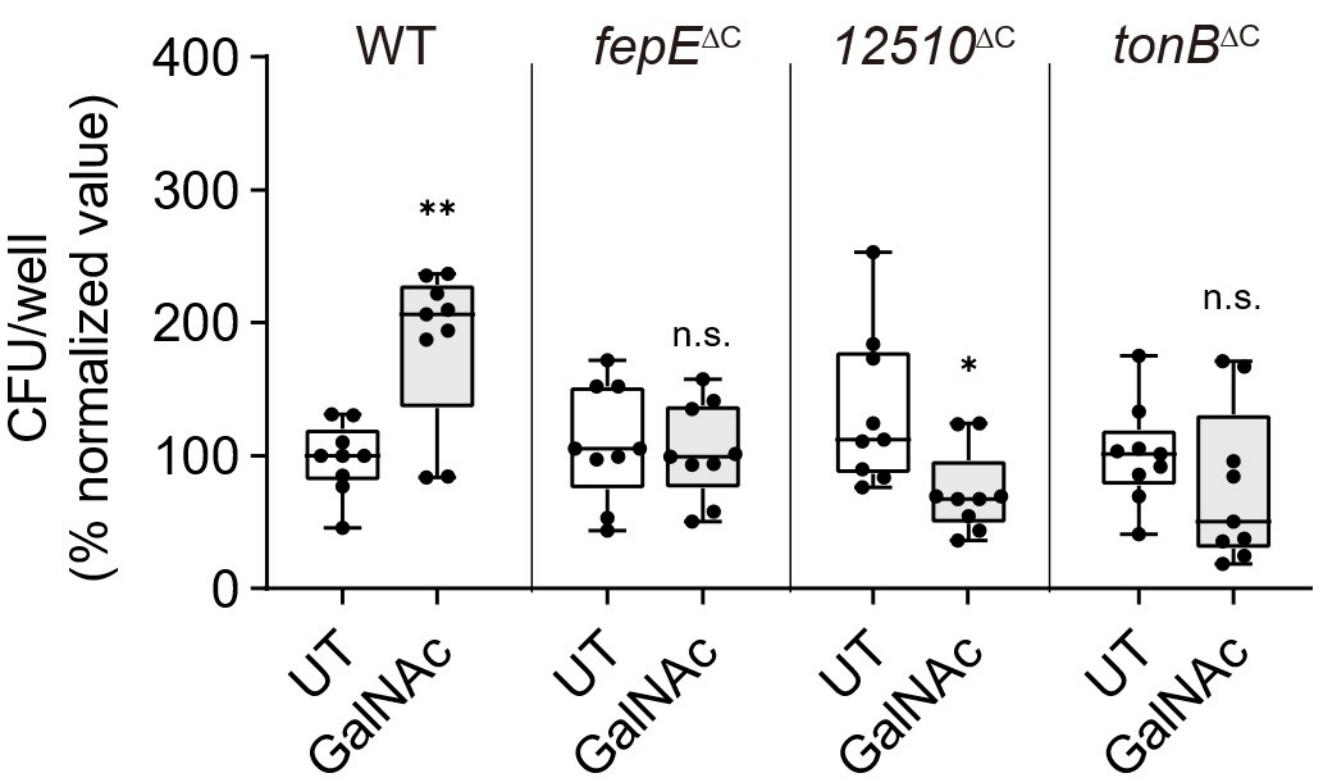

Figure S10, related to Fig. 3I-K. The periplasmic domains of FepE_STy, RS12510, and TonB are essential for GalNAc-mediated virulence regulation in *S*. Typhi. CFU analyses of STy recovered from infected human epithelial cells at 2 h post-infection. WT STy, STy*fepE*$^{\Delta C}$, STy*RS12510*$^{\Delta C}$, and STy*tonB*$^{\Delta C}$ were cultured in buffered LB.3 with or without GalNAc. Two–tailed unpaired t-tests between UT and GalNAc samples were performed. *P<0.05; **P < 0.01; n.s., not significant.

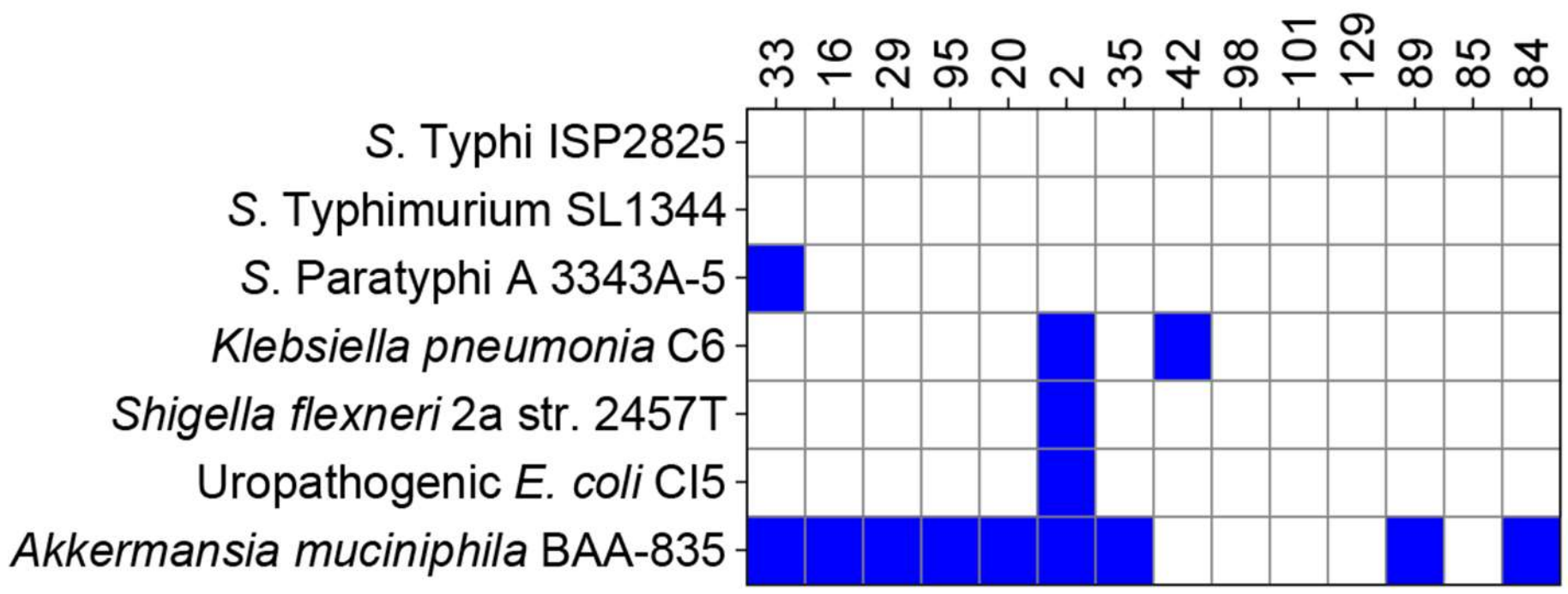

Figure S11, related to Fig. 4H. Heatmap summarizing mucin-associated GHs in *Salmonella* and other bacteria. The presence or absence of mucin-associated GHs was evaluated in *S.* Typhi ISP2825, *S.* Typhimurium SL1344, *S.* Paratyphi A 3343A-5, *Klebsiella pneumoniae* C6, *Shigella flexneri* 2a strain 2457T, Uropathogenic *E. coli* CI5, and *Akkermansia muciniphila* BAA-83533, using the Carbohydrate-Active enZYme Database (CAZy) (http://www.cazy.org/). The presence of each gene copy is shown in blue, and its absence in white.

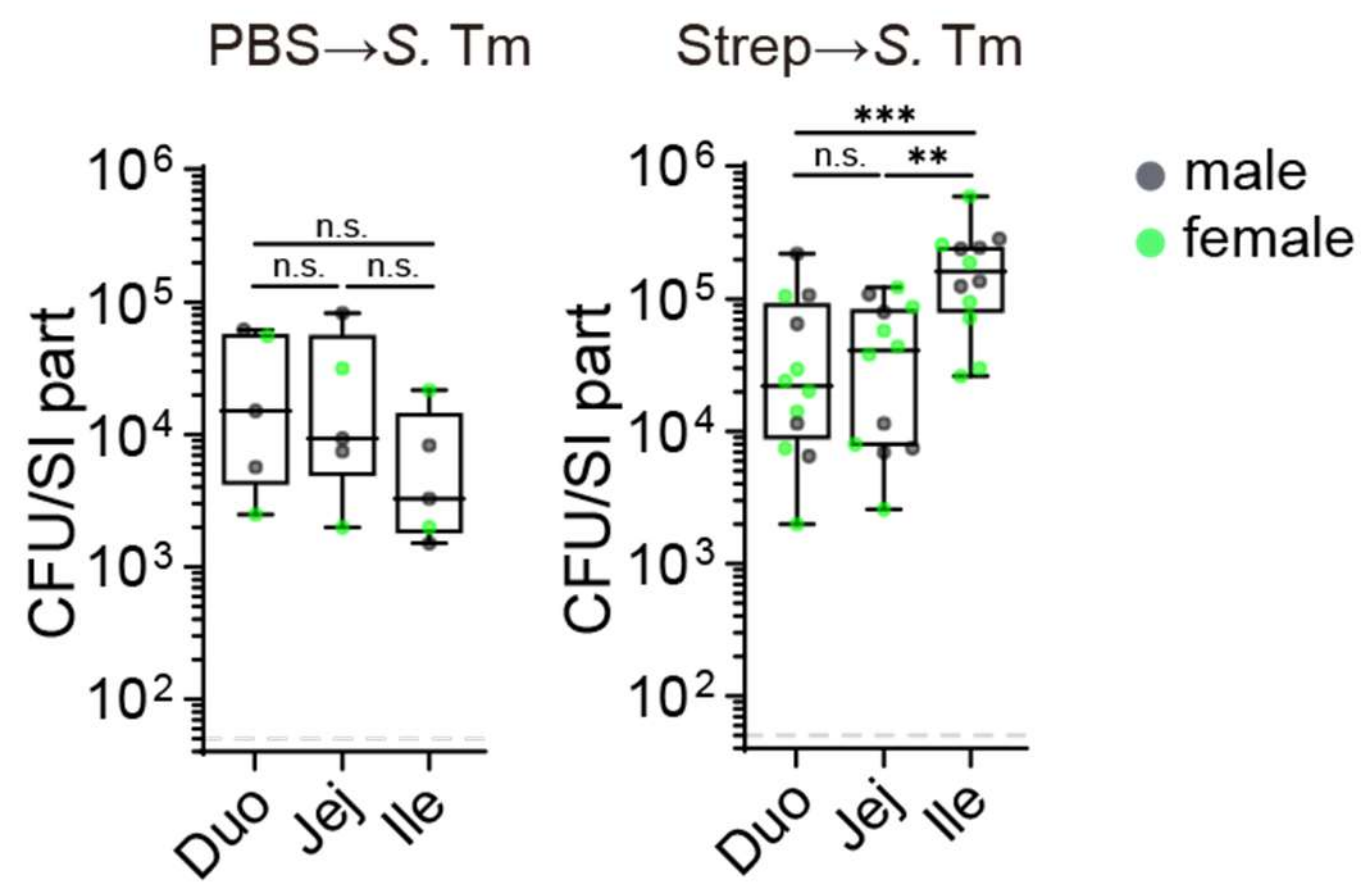

Figure S12, related to Fig. 4H. *S.* Tm exhibits mild ileum preference in streptomycin-pretreated mice. CFU data from 1-day post-infection. Intestinal samples from PBS-treated (left) or streptomycin-pretreated (right) Cmah null mice infected with *S.* Typhimurium. Two-tailed paired t-tests were used. **P<0.01; ***P < 0.001; n.s., not significant.

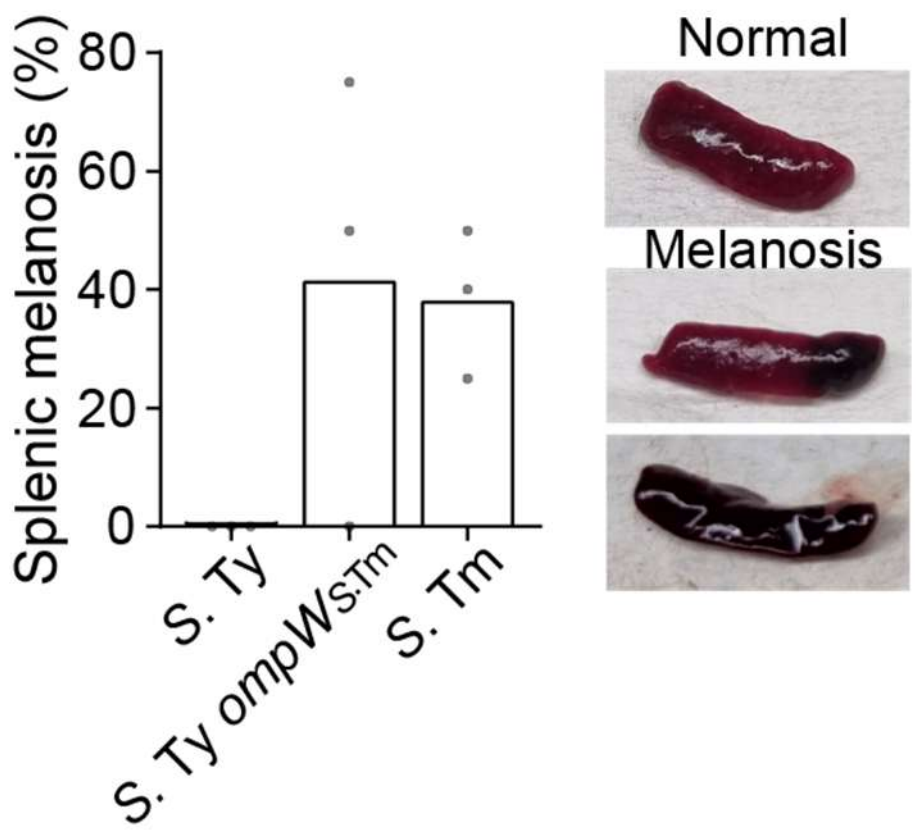

Figure S13, related to Fig. 5B. Splenic melanosis was frequently observed in mice infected with STy expressing OmpW$_{STm}$. Cmah null mice were orally infected for 3 days with STy, STy *ompW*$_{STm}$, or STm. The frequency of splenic melanosis from three independent experiments is shown (left), along with representative photographs (right). Each dot represents the percentage of splenic melanosis from 3 to 5 mice per experiment (left).

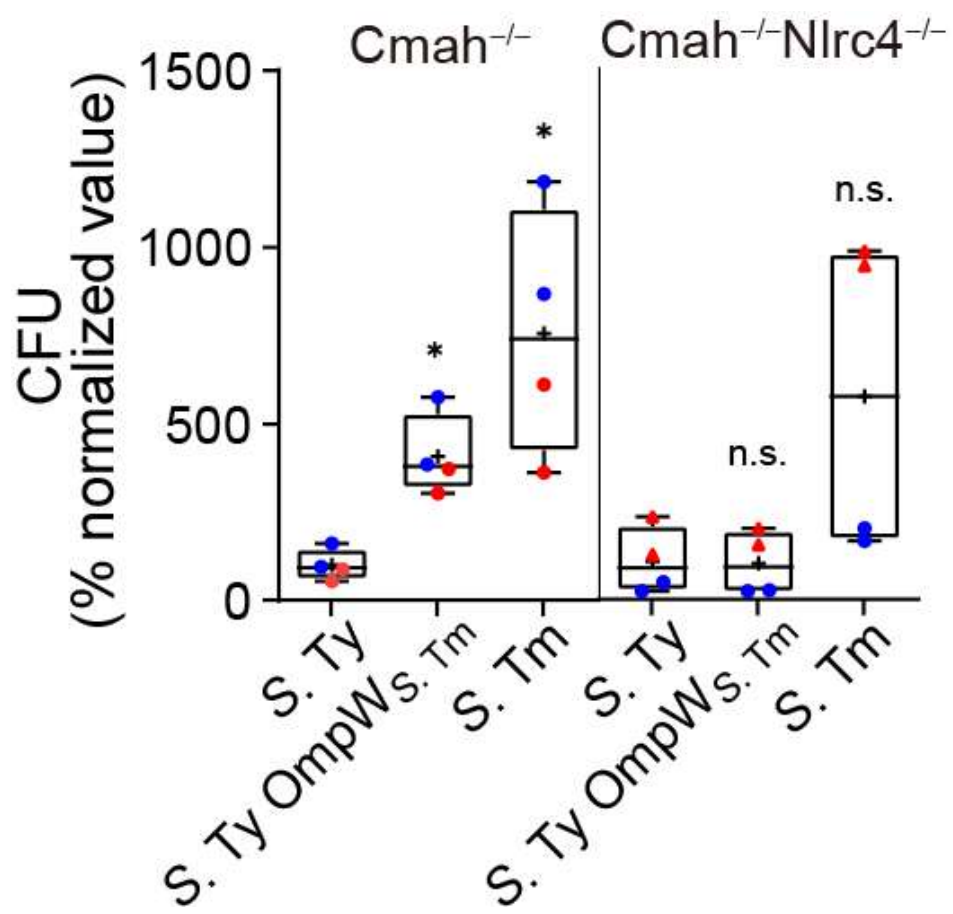

Figure S14, related to Fig. 5D. Nlrc4 in intestinal epithelial cells is crucial for OmpW-mediated regulation of STy virulence during the early stage of STy infection. CFU data from primary intestinal epithelial cells from Cmah null or Nlrc4 null/Cmah null mice infected with STy, STy $OmpW_{STm}$, or STm. CFU values were normalized to those from intestinal epithelial cells of Cmah null mice infected with WT STy. Two technical replicates from two mouse samples are shown in red and blue, respectively. Two-tailed Mann-Whitney U tests were performed relative to WT STy-infected samples. *P<0.05. n.s., not significant.

Table S4, related to Methods. Oligonucleotides used in this study.

	Name	Sequence (5' → 3')
Mouse genotyping	Muc2 common-F	TCCACATTATCACCTTGAC
	Muc2 WT-R	AGGGAATCGGTAGACATC
	Muc2 KO-R	GGATTGGGAAGACAATAG
	Nlrc4-F	CAGGTCACAGAAGAAGACCTGAATG
	Nlrc4-R	CACCTGGACTCCTGGATTTGG
	Cmah common-F	TCCCAGACCAGGAGGAGTTA
	Cmah WT-R	CTTCCAGTTGTGCTTTGTGC
	Cmah MT-R	GTCAGGAAACAGCACCAACA
RT-qPCR	*flhD*_F	TCATGCCCTTTTCTTACGCG
	*flhD*_R	GAGATGGCAAACACACTGGG
	*fliZ*_F	CGGGAATATGTCGTGCGTTT
	*fliZ*_R	ATTTCTGTCTGGGCGCAATC
	*fliA*_F	TGGTAAATGCCGTTCCTTGC
	*fliA*_R	CTGTGGCAGCGTTATGTACC
	*flgM*_F	GCTTCGACGCGTTCCATATT
	*flgM*_R	GAAACCCGTTAGCACTGTCC
	*fljB*_F	TTTCGGGGTGTAGGCATCTT
	*fljB*_R	ATCCAGGCTGAAATCACCCA
	*mcpC*_F	GCATTTCGGCGTTACTGGAT
	*mcpC*_R	GCGTCCTGAACAGCGATAAG
	*hilD*_F	TTTCTGCGCTTTCTCTGTGG
	*hilD*_R	GTGACGAACCTGGGATGTTG
	*hilA*_F	AAACCGATAACCCTGTCCGT
	*hilA*_R	TCCGCCAAAAGAATATGCCG
	*spaO*_F	GGAATGTGGGTTCGGTTGAG
	*spaO*_R	CAACAATTTCCCTTCCGGCA
	*sipB*_F	GCTGATTGGCAAGGCGATTA
	*sipB*_R	CGTTTCGCCCATCATTTTGC
	*sopB*_F	ACGTTAGAACCGGGTCTTGT
	*sopB*_R	GCTCGCCCGGAAATTATTGT
	*sopE*_F	TGCAACACACTTTCACCGAG
	*sopE*_R	AGGCGCTATGTTGATCCCTT
	*rtsA*_F	GGTTTTGTTAATGGGGCCGA
	*rtsA*_R	GACATCGTCCGGGGAGTATA
	*rrsA*_F	GAAATGCGTAGAGATCTGGAGG
	*rrsA*_R	CACAACCTCCAAGTAGACATCG

STy rRNA depletion	STy oligo 1	ACCGTTCGACTTGCATGTGTTAGGCCTGCCGCCAGCGTTC
	STy oligo 2	ATCAGGCAGTTTCCCAGACATTACTCACCCGTCCGCCACT
	STy oligo 3	GAAGGTCCCCCTCTTTGGTCTTGCGACGTTATGCGGTATT
	STy oligo 4	GTCGCCTTGGTGAGCCGTTACCTCACCAACAAGCTAATCC
	STy oligo 5	CGTAGGAGTCTGGACCGTGTCTCAGTTCCAGTGTGGCTG
	STy oligo 6	CTTCTTCATACACGTGGCATGGCTGCATCAGGCTTGCGCC
	STy oligo 7	CGTCAATTGCTGCGGTTATTAACCACAACACCTTCCTCCC
	STy oligo 8	TTAACGCTTGCACCCTCCGTATTACCGCGGCTGCTGGCAC
	STy oligo 9	GGTTGAGCCCGGGGATTTCACATCCGACTTGACAGACCGC
	STy oligo 10	TCACCGCTACACCTGGAATTCTACCCCCCTCTACAAGACT
	STy oligo 11	GGGCCGCCTTCGCCACCGGTATTCCTCCAGATCTCTACG
	STy oligo 12	TGGACTACCAGGGTATCTAATCCTGTTTGCTCCCCACGCT
	STy oligo 13	CTACTTAACGCGTTAGCTCCGGAAGCCACGCCTCAAGGGC
	STy oligo 14	CTCCACCGCTTGTGCGGGCCCCCGTCAATTCATTTGAGTT
	STy oligo 15	AAGTTCTGTGGATGTCAAGACCAGGTAAGGTTCTTCGCGT
	STy oligo 16	ACAACACGAGCTGACGACAGCCATGCAGCACCTGTCTCAC
	STy oligo 17	AGTTCCCGGCCGGACCGCTGGCAACAAAGGATAAGGGTTG
	STy oligo 18	GGTCGTAAGGGCCATGATGACTTGACGTCATCCCCACCTT
	STy oligo 19	TTATGAGGTCCGCTTGCTCTCGCGAGGTCGCTTCTCTTTG
	STy oligo 20	TACTAGCGATTCCGACTTCATGGAGTCGAGTTGCAGACTC
	STy oligo 21	CTCCCATGGTGTGACGGGCGGTGTGTACAAGGCCCGGGAA
	STy oligo 22	CCCCAGTCATGAATCACAAAGTGGTAAGCGCCCCCCCGAA
	STy oligo 23	TAAGGAGGTGATCCAACCGCAGGTTCCCCTACGGTTACC
	STy oligo 24	CGCAGATTAGCACGCCCTTCATCGCCTCTGACTGCCAGGG
	STy oligo 25	GAATCACACTGGGTTTCCCCATTCGGGTATCGCCGGTTAT
	STy oligo 26	GGGTACTTAGATGTTTCAGTTCCCCCGGTTCGCCTCATTA
	STy oligo 27	CCTCCCCGTTCGCTCGCCGCTACTGGGGGAATCTCGGTTG
	STy oligo 28	CTGTCACCCTGTATCGCGCGCCTTTCCAGACGCTTCCAC
	STy oligo 29	ATTCAGACAGGATACCACGTGTCCCGCCCTACTCATCGAG
	STy oligo 30	CTTTCCCTCACGGTACTGGTTCACTATCGGTCAGTCAGGA
	STy oligo 31	GGGGCTCCCACTGCTTGTACGTACACGGTTTCAGGTTCTT
	STy oligo 32	TCGCTTTCGCTCCGTTAACCGTCTCCTCAGCAAAGGATGC
	STy oligo 33	TTATACAAAAGGTACGCAGTCACCCCACCACTAAGCCCCC
	STy oligo 34	GTTAAGACTCGGTTTCCCTCCGGCTCCCCTATACAGTTAA
	STy oligo 35	TACCCAACCTTCAACCTGCCCATGGCTAGATCACCGGGTT
	STy oligo 36	CCTTTCACCCCCAGCCACAGGTCATCCGCTAATTTTTCAA
	STy oligo 37	GGCGCTACCTAAATAGCTTTCGGGGAGAACCAGCTATCTC
	STy oligo 38	GCATCGGGTTGGTAAGCCGGGATGGCCCCCTAGCCGAAAC
	STy oligo 39	CCCTCTTCACGACGGACATTAGCACCCGCCGTGTGTCTCC

	STy oligo 40	GGGCCTTCCCACATCGTTTCCCACTTAACCATGACTTTG
	STy oligo 41	TTAAATGATGGCTGCTTCTAAGCCAACATCCTGGCTGTCT
	STy oligo 42	TCGGTGCATGGTTTAGCCCCGTTACATCTTCCGCGCAGGC
	STy oligo 43	TCACAGCACACCTTCACAGGCTTACAGAACGCTCCCCTAC
	STy oligo 44	CGAGCGGGCTTTTCACCCGCTTTATCGTTACTTATGTCAG
	STy oligo 45	CGGCCTCGCCTTAGGGGTCGACTCACCCTGCCCCGATTAA
	STy oligo 46	TTCTCCGTCCCCCCTTCGCAGTAACACCAAGTACGGGAAT
	STy oligo 47	CATACCTACACGCTTAAACCGGGACAACCGTCGCCCGGCC
	STy oligo 48	CTTCAGCACCGTAGTGCCTCGTCGTCACGCCTCAGTGTTA
	STy oligo 49	GGTTTGGGGTACGATTTGATGTTACCTGATGCTTAGAGGC
	STy oligo 50	CCATTTTGCCTAGTTCCTTCACCCGAGTTCTCTCAAGCGC
	STy oligo 51	GGCTGACTTCAGCTCCGTGAGTAAATCACTTCACCTACGT
	STy oligo 52	GTATACGTCCACTTTCGTGTTTGCACAGTGCTGTGTTTTT
	STy oligo 53	GCTTCGATCAGGAGCTTCGCTTGCGCTGACCCCATCAATT
	STy oligo 54	GGAATTTCGCTACCTTAGGACCGTTATAGTTACGGCCGCC
	STy oligo 55	CACTGAGTCTCGGGTGGAGACAGCCTGGCCATCATTACGC
	STy oligo 56	TATAGTAAAGGTTCACGGGGTCTTTCCGTCTTGCCGCGGG
	STy oligo 57	GGCGTCCACACTTCAAAGCCTCCCACCTATCCTACACAT
	STy oligo 58	CATTAAAGGGTGGTATTTCAAGGTCGGCTCCATGCAGACT
	STy oligo 59	CCGCCCCAGTCAAACTACCCACCAGACACTGTCCGCAACC
	STy oligo 60	GCACTAACCTCCTGATGTCCGACCAGGATTAGCCAACCTT
	STy oligo 61	CGGATCACTATGACCTGCTTTCGCACCTGCTCGCGCCGTC
	STy oligo 62	GGCGGTATCAGCCTGTTATCCCCGGAGTACCTTTTATCCG
	STy oligo 63	CCTACTTCAGCCCCAGGATGTGATGAGCCGACATCGAGGT
	STy oligo 64	AACTGTCTCACGACGTTCTAAACCCAGCTCGCGTACCACT
	STy oligo 65	CCCCCTCAGTTCTCCAGCGCCCACGGCAGATAGGGACCG
	STy oligo 66	CAGTGCCATTGGCATGACAACCCGAACACCAGTGATGCGT
	STy oligo 67	AACTCATCTCGGGGCAAGTTTCGTGCTTAGATGCTTTCAG
	STy oligo 68	CGCTTACACACCCGGCCTATCAACGTCGTCGTCTTCAACG
	STy oligo 69	AAGGTTAAGCCTCACGGTTCATTAGTACCGGTTAGCTCA
	STy oligo 70	CGGCGCTACGGCGTTTCACTTCTGAGTTCGGCATGGGGTC
	STy oligo 71	CTGGCAGTTCCCTACTCTCGCATGGGGAGACCCCACACT

Table S5, related to Methods. Strains and plasmids used in this study.

ID	Description	Ref
Strains		
Salmonella enterica Typhi		
SB2201	ISP2825 Wild type	[1]
SB2204	ISP2825 carrying pWSK-mCherry	This study
JS0294	ISP2825 Δ*galP*Δ*mglB*	This study
JS0296	ISP2825 Δ*nagE*Δ*manX*	This study
JS0264	ISP2825 $ompW_{STm}$	This study
JS0266	ISP2825 $ompF_{STm}$	This study
JS0268	ISP2825 $fljB_{STm}$	This study
JS0271	ISP2825 $lamB_{STm}$	This study
JS0275	ISP2825 $ompW_{STm}$ $ompF_{STm}$ $fljB_{STm}$ $lamB_{STm}$	This study
JS0276	ISP2825 $ompW^{K141N/T142A}$	This study
JS0277	ISP2825 $ompW^{H193Q}$	This study
JS0272	ISP2825 P*hilD*-lacZ	This study
JS0278	ISP2825 Δ*fepE*	This study
JS0279	ISP2825 Δ*RS12510*	This study
JS0280	ISP2825 Δ*RS17905*	This study
JS0281	ISP2825 Δ*fepE* P*hilD*-lacZ	This study
JS0282	ISP2825 Δ*RS12510* P*hilD*-lacZ	This study
JS0283	ISP2825 Δ*17905* P*hilD*-lacZ	This study
JS0284	ISP2825 $ompW_{STm}$ P*hilD*-lacZ ISP2825	This study
JS0285	ISP2825 Δ*fepE* carrying pWSK-$fepE^{short}$	This study
JS0286	ISP2825 Δ*fepE* carrying pWSK-*fepE*	This study
JS0287	ISP2825 $fepE^{+}$	This study
JS0288	ISP2825 Δ*RS12510* carrying pWSK-RS12510	This study
JS0289	ISP2825 Δ*tonB*	This study
JS0290	ISP2825 Δ*tonB* carrying pWSK-*tonB*	This study
JS0291	ISP2825 $ompW_{STm}$ carrying pWSK-mCherry	This study
JS0066	ISP2825 *tviE* P263S (Kan^R)	[2]
JS0292	ISP2825 $ompW_{STm}$ *tviE* P263S, Kan^R	This study
JS0298	ISP2825 $fepE^{\Delta C}$	This study
JS0299	ISP2825 $RS12510^{\Delta C}$	This study
JS0300	ISP2825 $tonB^{\Delta C}$	This study
Salmonella enterica Typhimurium		
JS0297	SL1344 carrying pWSK-mCherry	This study
JS0274	SL1344 $ompW_{STy}$ carrying pWSK-mCherry	This study
Escherichia coli		
pSB5022	BL21(DE3) carrying pET28a-pltB-pltA-cdtB-His_6	[3]
Plasmids		
pJS0521	pSB890-Δ*galP*	This study
pJS0522	pSB890-Δ*mglB*	This study
pJS0523	pSB890-Δ*nagE*	This study
pJS0524	pSB890-Δ*manX*	This study
pJS0466	pSB890-Δ*ompW*	This study
pJS0467	pSB890-Δ*ompF*	This study
pJS0468	pSB890-Δ*fljB*	This study
pJS0469	pSB890-Δ*lamB*	This study
pJS0470	pSB890-$ompW_{STm}$	This study
pJS0471	pSB890-$ompF_{STm}$	This study
pJS0472	pSB890-$fljB_{STm}$	This study
pJS0474	pSB890-$lamB_{STm}$	This study
pJS0484	pSB890-Δ*RS01450*	This study

pJS0487	pSB890- P*hilD*-LacZ	This study
pJS0525	pCat-mariner (Kan^R)	[4]
pJS0513	pSB890-Δ*fepE*	This study
pJS0514	pSB890-Δ*RS12510*	This study
pJS0515	pSB890-Δ*RS17905*	This study
pJS0516	pSB890-Δ*tonB*	This study
pJS0517	pWSK-PrpsM-*fepE*^{short}	This study
pJS0518	pWSK-PrpsM-*fepE*	This study
pJS0519	pWSK-PrpsM-*RS12510*	This study
pJS0520	pWSK-PrpsM-*tonB*	This study

Table S6. Related to Methods. Semi-qRT-PCR conditions and oligonucleotides used.

RT			
Steps	Temperature	Time	Cycles
Priming	25℃	5 min	1
Reverse transcription	42℃	60 min	1
RT Inactivation	95℃	1 min	1
Hold	12℃	Forever	
**PCR**			
Steps	Temperature	Time	Cycles
Initial Denaturation	94℃	5 min	1
Denaturation	94℃	30 sec	16s rRNA:20<br>*hilD*: 30
Annealing	60℃	30 sec	
Extension	72℃	30 sec	
Final extension	72℃	5 min	
Hold	12℃	Forever	

Oligonucleotides		
Target	Sequence (5'-3')	Product size
16s rRNA	Forward GAAATGCGTAGAGATCTGGAGG<br>Reverse CACAACCTCCAAGTAGACATCG	146bp
hilD	Forward TTTCTGCGCTTTCTCTGTGG<br>Reverse GTGACGAACCTGGGATGTTG	185bp

References

1. Galán, J.E., and Curtiss, R. (1991). Distribution of the *invA*, *-B*, *-C*, and *-D* Genes of *Salmonella typhimurium* among Other *Salmonella* Serovars: *invA* Mutants of *Salmonella typhi* Are Deficient for Entry into Mammalian Cells. Infect Immun *59*, 2901-2908. doi:10.1128/iai.59.9.2901-2908.1991.
2. Lee, G.Y., and Song, J. (2024). Single missense mutations in Vi capsule synthesis genes confer hypervirulence to *Salmonella* Typhi. Nat Commun *15*, 5258. 10.1038/s41467-024-49590-6.
3. Song, J., Gao, X., and Galan, J.E. (2013). Structure and function of the *Salmonella* Typhi chimaeric A_2B_5 typhoid toxin. Nature *499*, 350-354. 10.1038/nature12377.
4. Gao, B., Lara-Tejero, M., Lefebre, M., Goodman, A.L., and Galan, J.E. (2014). Novel components of the flagellar system in epsilonproteobacteria. MBio *5*, e01349-01314. 10.1128/mBio.01349-14.
